# Trace-Relating Compiler Correctness and Secure Compilation

**DOI:** 10.1007/978-3-030-44914-8_1

**Published:** 2020-04-18

**Authors:** Carmine Abate, Roberto Blanco, Ștefan Ciobâcă, Adrien Durier, Deepak Garg, Cătălin Hrițcu, Marco Patrignani, Éric Tanter, Jérémy Thibault

**Affiliations:** ETH Zurich, Zurich, Switzerland; 9 Inria Paris, Paris, France; 10 UAIC Iaşi, Iaşi, Romania; 11 MPI-SWS, Saarbrücken, Germany; 12 Stanford University, Stanford, USA; 13 CISPA, Saarbrücken, Germany; 14 University of Chile, Santiago, Chile

## Abstract

Compiler correctness is, in its simplest form, defined as the inclusion of the set of traces of the compiled program into the set of traces of the original program, which is equivalent to the preservation of all trace properties. Here traces collect, for instance, the externally observable events of each execution. This definition requires, however, the set of traces of the source and target languages to be exactly the same, which is not the case when the languages are far apart or when observations are fine-grained. To overcome this issue, we study a generalized compiler correctness definition, which uses source and target traces drawn from potentially different sets and connected by an arbitrary relation. We set out to understand what guarantees this generalized compiler correctness definition gives us when instantiated with a non-trivial relation on traces. When this trace relation is not equality, it is no longer possible to preserve the trace properties of the source program unchanged. Instead, we provide a generic characterization of the target trace property ensured by correctly compiling a program that satisfies a given source property, and dually, of the source trace property one is required to show in order to obtain a certain target property for the compiled code. We show that this view on compiler correctness can naturally account for undefined behavior, resource exhaustion, different source and target values, side-channels, and various abstraction mismatches. Finally, we show that the same generalization also applies to many secure compilation definitions, which characterize the protection of a compiled program against linked adversarial code.

### 1 Introduction

Compiler correctness is an old idea [37, 40, 41] that has seen a significant revival in recent times. This new wave was started by the creation of the CompCert verified C compiler [33] and continued by the proposal of many significant extensions and variants of CompCert [8, 9, 12, 23, 29, 30, 42, 52, 56, 57, 61] and the success of many other milestone compiler verification projects, including Vellvm [64], Pilsner [45], CakeML [58], CertiCoq [4], etc. Yet, even for these verified compilers, the precise statement of correctness matters. Since proof assistants are used to conduct the verification, an external observer does not have to understand the proofs in order to trust them, but one still has to deeply understand the statement that was proved. And this is true not just for correct compilation, but also for secure compilation, which is the more recent idea that our compilation chains should do more to also ensure security of our programs [3, 26].

**Basic Compiler Correctness.** The gold standard for compiler correctness is *semantic preservation*, which intuitively says that the semantics of a compiled program (in the target language) is compatible with the semantics of the original program (in the source language). For practical verified compilers, such as CompCert [33] and CakeML [58], semantic preservation is stated extrinsically, by referring to *traces*. In these two settings, a trace is an ordered sequence of events—such as inputs from and outputs to an external environment—that are produced by the execution of a program.

A basic definition of compiler correctness can be given by the set inclusion of the traces of the compiled program into the traces of the original program. Formally [33]:

**Definition 1.1 (Basic Compiler Correctness (CC)).** *A compiler* $\downarrow$ *is* correct *iff*

$$\forall \mathsf{W}\ t.\ \mathsf{W}{\downarrow} \rightsquigarrow t \Rightarrow \mathsf{W} \rightsquigarrow t.$$

This definition says that for any whole[1] source program W, if we compile it (denoted $\mathsf{W}{\downarrow}$), execute it with respect to the semantics of the target language, and observe a trace $t$, then the original W can produce *the same* trace $t$ with respect to the semantics of the source language.[2] This definition is simple and easy to understand, since it only references a few familiar concepts: a compiler between a source and a target language, each equipped with a trace-producing semantics (usually nondeterministic).

**Beyond Basic Compiler Correctness.** This basic compiler correctness definition assumes that any trace produced by a compiled program can be produced by the source program. This is a very strict requirement, and in particular implies that the source and target traces are drawn from the same set and that the same source trace corresponds to a given target trace. These assumptions are often too strong, and hence in practice verified compiler efforts use different formulations of compiler correctness:

**CompCert [33]** The original compiler correctness theorem of CompCert [33] can be seen as an instance of basic compiler correctness, but it does not provide any guarantees for programs that can exhibit undefined behavior [53]. As allowed by the C standard, such unsafe programs are not even considered to be in the source language, so are not quantified over. This has important practical implications, since undefined behavior often leads to exploitable security vulnerabilities [13, 24, 25] and serious confusion even among experienced C and C++ developers [32, 53, 59, 60]. As such, since 2010, CompCert provides an additional top-level correctness theorem[3] that better accounts for the presence of unsafe programs by providing guarantees for them up to the point when they encounter undefined behavior [53]. This new theorem goes beyond the basic correctness definition above, as a target trace need only correspond to a source trace *up to the occurrence* of undefined behavior in the source trace.

**CakeML [58]** Compiler correctness for CakeML accounts for memory exhaustion in target executions. Crucially, memory exhaustion events cannot occur in source traces, only in target traces. Hence, dually to CompCert, compiler correctness only requires source and target traces to coincide up to the occurrence of a memory exhaustion event in the target trace.

[1] For simplicity, for now we ignore separate compilation and linking, returning to it in §5.

[2] Typesetting convention [47]: we use a blue, sans-serif font for source elements, an **orange**, **bold** font for **target** ones and a *black*, *italic* font for elements common to both languages.

[3] Stated at the top of the CompCert file `driver/Complements.v` and discussed by Regehr [53].

**Trace-Relating Compiler Correctness.** Generalized formalizations of compiler correctness like the ones above can be naturally expressed as instances of a uniform definition, which we call *trace-relating compiler correctness*. This generalizes basic compiler correctness by (a) considering that source and target traces belong to *possibly distinct* sets $\mathsf{Trace_S}$ and $\mathbf{Trace_T}$, and (b) being parameterized by an arbitrary *trace relation* $\sim$.

**Definition 1.2 (Trace-Relating Compiler Correctness ($\mathsf{CC}^{\sim}$)).** *A compiler* $\downarrow$ *is* correct *with respect to a trace relation* $\sim \subseteq \mathsf{Trace_S} \times \mathbf{Trace_T}$ *iff*

$$\forall \mathsf{W}. \forall \mathbf{t}.\ \mathsf{W}\downarrow \rightsquigarrow \mathbf{t} \Rightarrow \exists \mathsf{s} \sim \mathbf{t}.\ \mathsf{W} \rightsquigarrow \mathsf{s}.$$

This definition requires that, for any target trace $\mathbf{t}$ produced by the compiled program $\mathsf{W}\downarrow$, there exist a source trace $\mathsf{s}$ that can be produced by the original program $\mathsf{W}$ and is *related* to $\mathbf{t}$ according to $\sim$ (i.e., $\mathsf{s} \sim \mathbf{t}$). By choosing the trace relation appropriately, one can recover the different notions of compiler correctness presented above:

**Basic CC** Take $\mathsf{s} \sim \mathbf{t}$ to be $\mathsf{s} = \mathbf{t}$. Trivially, the basic CC of Definition 1.1 is $\mathsf{CC}^{=}$.

**CompCert** Undefined behavior is modeled in CompCert as a trace-terminating event *Goes_wrong* that can occur in any of its languages (source, target, and all intermediate languages), so for a given phase (or composition thereof), we have $\mathsf{Trace_S} = \mathbf{Trace_T}$. Nevertheless, the relation between source and target traces with which to instantiate $\mathsf{CC}^{\sim}$ to obtain CompCert's current theorem is:

$$\mathsf{s} \sim \mathbf{t} \quad \equiv \quad \mathsf{s} = \mathbf{t} \vee (\exists m \leq \mathbf{t}.\ \mathsf{s} = m \cdot Goes\_wrong).$$

A compiler satisfying $\mathsf{CC}^{\sim}$ for this trace relation can turn a source trace ending in undefined behavior $m \cdot Goes\_wrong$ (where "$\cdot$" is concatenation) either into the same trace in the target (first disjunct), or into a target trace that starts with the prefix $m$ but then continues *arbitrarily* (second disjunct, "$\leq$" is the prefix relation).

**CakeML** Here, target traces are sequences of symbols from an alphabet $\mathbf{\Sigma_T}$ that has a specific trace-terminating event, $\mathbf{Resource\_limit\_hit}$, which is not available in the source alphabet $\mathsf{\Sigma_S}$ (i.e., $\mathbf{\Sigma_T} = \mathsf{\Sigma_S} \cup \{\mathbf{Resource\_limit\_hit}\}$. Then, the compiler correctness theorem of CakeML can be obtained by instantiating $\mathsf{CC}^{\sim}$ with the following $\sim$ relation:

$$\mathsf{s} \sim \mathbf{t} \quad \equiv \quad \mathsf{s} = \mathbf{t} \vee (\exists m.\ m \leq \mathsf{s}.\ \mathbf{t} = m \cdot \mathbf{Resource\_limit\_hit}).$$

The resulting $\mathsf{CC}^{\sim}$ instance relates a target trace ending in $\mathbf{Resource\_limit\_hit}$ after executing $m$ to a source trace that first produces $m$ and then continues in a way given by the semantics of the source program.

Beyond undefined behavior and resource exhaustion, there are many other practical uses for $\mathsf{CC}^{\sim}$: in this paper we show that it also accounts for differences between source and target values, for a single source output being turned into a series of target outputs, and for side-channels.

On the flip side, the compiler correctness statement and its implications can be more difficult to understand for $\mathsf{CC}^{\sim}$ than for $\mathsf{CC}^{=}$. The full implications of choosing a particular $\sim$ relation can be subtle. In fact, using a bad relation can make the compiler correctness statement trivial or unexpected. For instance, it should be easy to see that if one uses the total relation, which relates all source traces to all target ones, the $\mathsf{CC}^{\sim}$ property holds for every compiler, yet it might take one a bit more effort to understand that the same is true even for the following relation:

$$\mathsf{s} \sim \mathbf{t} \quad \equiv \quad \exists \mathsf{W}. \mathsf{W} \rightsquigarrow \mathsf{s} \wedge \mathsf{W}\downarrow \rightsquigarrow \mathbf{t}.$$

**Reasoning About Trace Properties.** To understand more about a particular $\mathsf{CC}^{\sim}$ instance, we propose to also look at how it preserves *trace properties*—defined as sets of allowed traces [31]—from the source to the target. For instance, it is well known that $\mathsf{CC}^{=}$ is equivalent to the preservation of all trace properties (where $W \models \pi$ reads "$W$ satisfies $\pi$" and stands for $\forall t.\ W \rightsquigarrow t \Rightarrow t \in \pi$):

$$\mathsf{CC}^{=} \quad \equiv \quad \forall \pi \in 2^{\text{Trace}}\, \forall \mathsf{W}.\ \mathsf{W} \models \pi \Rightarrow \mathsf{W}{\downarrow} \models \pi.$$

However, to the best of our knowledge, similar results have not been formulated for trace relations beyond equality, when it is no longer possible to preserve the trace properties of the source program unchanged. For trace-relating compiler correctness, where source and target traces can be drawn from different sets and related by an arbitrary trace relation, there are two crucial questions to ask:

1. For a source trace property $\pi_{\mathsf{S}}$ of a program—established for instance by formal verification—what is the strongest target property that any $\mathsf{CC}^{\sim}$ compiler is guaranteed to ensure for the produced target program?
2. For a target trace property $\pi_{\mathbf{T}}$, what is the weakest source property we need to show of the original source program to obtain $\pi_{\mathbf{T}}$ for the result of any $\mathsf{CC}^{\sim}$ compiler?

Far from being mere hypothetical questions, they can help the developer of a verified compiler to better understand the compiler correctness theorem they are proving, and we expect that any user of such a compiler will need to ask either one or the other if they are to make use of that theorem. In this work we provide a simple and natural answer to these questions, for any instance of $\mathsf{CC}^{\sim}$. Building upon a bijection between relations and Galois connections [5, 20, 43], we observe that any trace relation $\sim$ corresponds to two *property mappings* $\tilde{\tau}$ and $\tilde{\sigma}$, which are functions mapping source properties to target ones ($\tilde{\tau}$ standing for "to target") and target properties to source ones ($\tilde{\sigma}$ standing for "to source"):

$$\tilde{\tau}(\pi_{\mathsf{S}}) = \{\mathbf{t} \mid \exists \mathsf{s}.\ \mathsf{s} \sim \mathbf{t} \wedge \mathsf{s} \in \pi_{\mathsf{S}}\}\,; \qquad \tilde{\sigma}(\pi_{\mathbf{T}}) = \{\mathsf{s} \mid \forall \mathbf{t}.\ \mathsf{s} \sim \mathbf{t} \Rightarrow \mathbf{t} \in \pi_{\mathbf{T}}\}\,.$$

The *existential image* of $\sim$, $\tilde{\tau}$, answers the first question above by mapping a given source property $\pi_{\mathsf{S}}$ to the target property that contains all target traces for which *there exists a related source trace* that satisfies $\pi_{\mathsf{S}}$. Dually, the *universal image* of $\sim$, $\tilde{\sigma}$, answers the second question by mapping a given target property $\pi_{\mathbf{T}}$ to the source property that contains all source traces for which *all related target traces* satisfy $\pi_{\mathbf{T}}$. We introduce two new correct compilation definitions in terms of *trace property preservation* ($\mathsf{TP}$): $\mathsf{TP}^{\tilde{\tau}}$ quantifies over all source trace properties and uses $\tilde{\tau}$ to obtain the corresponding target properties. $\mathsf{TP}^{\tilde{\sigma}}$ quantifies over all target trace properties and uses $\tilde{\sigma}$ to obtain the corresponding source properties. We prove that these two definitions are equivalent to $\mathsf{CC}^{\sim}$, yielding a novel trinitarian view of compiler correctness (Figure 1).

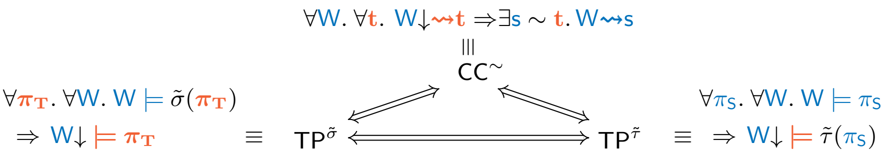

Fig. 1: The equivalent compiler correctness definitions forming our trinitarian view.

**Contributions.**

- We propose a new trinitarian view of compiler correctness that accounts for non-trivial trace relations. While, as discussed above, specific instances of the $\mathsf{CC}^{\sim}$ definition have already been used in practice, we seem to be the first to propose assessing the meaningfulness of $\mathsf{CC}^{\sim}$ instances in terms of how properties are preserved between the source and the target, and in particular by looking at the property mappings $\tilde{\sigma}$ and $\tilde{\tau}$ induced by the trace relation $\sim$. We prove that $\mathsf{CC}^{\sim}$, $\mathsf{TP}^{\tilde{\sigma}}$, and $\mathsf{TP}^{\tilde{\tau}}$ are equivalent for any trace relation (§2.2), as illustrated in Figure 1. In the opposite direction, we show that for every trace relation corresponding to a given Galois connection [20], an analogous equivalence holds. Finally, we extend these results (§2.3) from the preservation of trace properties to the larger class of subset-closed hyperproperties (e.g., noninterference).
- We use $\mathsf{CC}^{\sim}$ compilers of various complexities to illustrate that our view on compiler correctness naturally accounts for undefined behavior (§3.1), resource exhaustion (§3.2), different source and target values (§3.3), and differences in the granularity of data and observable events (§3.4). We expect these ideas to apply to any other discrepancies between source and target traces. For each compiler we show how to choose the relation between source and target traces and how the induced property mappings preserve interesting trace properties and subset-closed hyperproperties. We look at the way particular $\tilde{\sigma}$ and $\tilde{\tau}$ work on different kinds of properties and how the produced properties can be expressed for different kinds of traces.
- We analyze the impact of correct compilation on noninterference [22], showing what can still be preserved (and thus also what is lost) when target observations are finer than source ones, e.g., side-channel observations (§4). We formalize the guarantee obtained by correct compilation of a noninterfering program as *abstract noninterference* [21], a weakening of target noninterference. Dually, we identify a family of declassifications of target noninterference for which source reasoning is possible.
- Finally, we show that the trinitarian view also extends to a large class of *secure compilation* definitions [2], formally characterizing the protection of the compiled program against linked adversarial code (§5). For each secure compilation definition we again propose both a property-free characterization in the style of $\mathsf{CC}^{\sim}$, and two characterizations in terms of preserving a class of source or target properties satisfied against arbitrary adversarial contexts. The additional quantification over contexts allows for finer distinctions when considering different property classes, so we study mapping classes not only of trace properties and hyperproperties, but also of relational hyperproperties [2]. An example secure compiler accounting for a target that can produce additional trace events that are not possible in the source illustrates this approach.

The paper closes with discussions of related (§6) and future work (§7). An online appendix contains omitted technical details: `https://arxiv.org/abs/1907.05320`.

The traces considered in our examples are structured, usually as sequences of events. We notice however that unless explicitly mentioned, all our definitions and results are more general and make no assumption whatsoever about the structure of traces. Most of the theorems formally or informally mentioned in the paper were mechanized in the Coq proof assistant and are marked with . This development has around 10k lines of code, is described in the online appendix, and is available at the following address: `https://github.com/secure-compilation/different_traces`.

## 2 Trace-Relating Compiler Correctness

In this section, we start by generalizing the trace property preservation definitions at the end of the introduction to $\mathsf{TP}^{\sigma}$ and $\mathsf{TP}^{\tau}$, which depend on two *arbitrary* mappings $\sigma$ and $\tau$ (§2.1). We prove that, whenever $\sigma$ and $\tau$ form a Galois connection, $\mathsf{TP}^{\sigma}$ and $\mathsf{TP}^{\tau}$ are equivalent (Theorem 2.4). We then exploit a bijective correspondence between trace relations and Galois connections to close the trinitarian view (§2.2), with two main benefits: first, it helps us assess the meaningfulness of a given trace relation by looking at the property mappings it induces; second, it allows us to construct new compiler correctness definitions starting from a desired mapping of properties. Finally, we generalize the classic result that compiler correctness (i.e., $\mathsf{CC}^{=}$) is enough to preserve not just trace properties but also all subset-closed hyperproperties [14]. For this, we show that $\mathsf{CC}^{\sim}$ is also equivalent to subset-closed hyperproperty preservation, for which we also define both a version in terms of $\tilde{\sigma}$ and a version in terms of $\tilde{\tau}$ (§2.3).

### 2.1 Property Mappings

As explained in §1, trace-relating compiler correctness $\mathsf{CC}^{\sim}$, by itself, lacks a crisp description of which trace properties are preserved by compilation. Since even the syntax of traces can differ between source and target, one can either look at trace properties of the source (but then one needs to interpret them in the target), or at trace properties of the target (but then one needs to interpret them in the source). Formally we need two property mappings, $\tau : 2^{\mathsf{Trace_S}} \to 2^{\mathbf{Trace_T}}$ and $\sigma : 2^{\mathbf{Trace_T}} \to 2^{\mathsf{Trace_S}}$, which lead us to the following generalization of trace property preservation (TP).

**Definition 2.1** ($\mathsf{TP}^{\sigma}$ **and** $\mathsf{TP}^{\tau}$)**.** *Given two property mappings,* $\tau : 2^{\mathsf{Trace_S}} \to 2^{\mathbf{Trace_T}}$ *and* $\sigma : 2^{\mathbf{Trace_T}} \to 2^{\mathsf{Trace_S}}$*, for a compilation chain* $\cdot\!\downarrow$ *we define:*

$$\mathsf{TP}^{\tau} \equiv \forall \pi_{\mathsf{S}}.\, \forall \mathsf{W}.\, \mathsf{W} \models \pi_{\mathsf{S}} \Rightarrow \mathsf{W}\!\downarrow\, \models \tau(\pi_{\mathsf{S}}); \quad \mathsf{TP}^{\sigma} \equiv \forall \boldsymbol{\pi_{\mathrm{T}}}.\, \forall \mathsf{W}.\, \mathsf{W} \models \sigma(\boldsymbol{\pi_{\mathrm{T}}}) \Rightarrow \mathsf{W}\!\downarrow\, \models \boldsymbol{\pi_{\mathrm{T}}}.$$

For an arbitrary source program $\mathsf{W}$, $\tau$ interprets a source property $\pi_{\mathsf{S}}$ as the *target guarantee* for $\mathsf{W}\!\downarrow$. Dually, $\sigma$ defines a *source obligation* sufficient for the satisfaction of a target property $\boldsymbol{\pi_{\mathrm{T}}}$ after compilation. Ideally:

- Given $\boldsymbol{\pi_{\mathrm{T}}}$, the target interpretation of the source obligation $\sigma(\boldsymbol{\pi_{\mathrm{T}}})$ should actually guarantee that $\boldsymbol{\pi_{\mathrm{T}}}$ holds, i.e., $\tau(\sigma(\boldsymbol{\pi_{\mathrm{T}}})) \subseteq \boldsymbol{\pi_{\mathrm{T}}}$;
- Dually for $\pi_{\mathsf{S}}$, we would not want the source obligation for $\tau(\pi_{\mathsf{S}})$ to be harder than $\pi_{\mathsf{S}}$ itself, i.e., $\sigma(\tau(\pi_{\mathsf{S}})) \supseteq \pi_{\mathsf{S}}$.

These requirements are satisfied when the two maps form a *Galois connection* between the posets of source and target properties ordered by inclusion. We briefly recall the definition and the characteristic property of Galois connections [16, 38].

**Definition 2.2 (Galois connection).** *Let* $(X, \preceq)$ *and* $(Y, \sqsubseteq)$ *be two posets. A pair of maps,* $\alpha : X \to Y, \gamma : Y \to X$ *is a Galois connection iff it satisfies the* adjunction law*:* $\forall x \in X.\, \forall y \in Y.\, \alpha(x) \sqsubseteq y \iff x \preceq \gamma(y)$*.* $\alpha$ *(resp.* $\gamma$*) is the lower (upper) adjoint or abstraction (concretization) function and* $Y$ $(X)$ *the abstract (concrete) domain.*

We will often write $\alpha : (X, \preceq) \leftrightarrows (Y, \sqsubseteq) : \gamma$ to denote a Galois connection, or simply $\alpha : X \leftrightarrows Y : \gamma$, or even $\alpha \leftrightarrows \gamma$ when the involved posets are clear from context.

**Lemma 2.3 (Characteristic property of Galois connections).** *If* $\alpha{:}(X, \preceq) \leftrightarrows (Y, \sqsubseteq){:}\gamma$ *is a Galois connection, then* $\alpha, \gamma$ *are monotone and they satisfy these properties:*

$$i)\quad \forall x \in X.\, x \preceq \gamma(\alpha(x)); \qquad\qquad ii)\quad \forall y \in Y.\, \alpha(\gamma(y)) \sqsubseteq y.$$

*If* $X, Y$ *are complete lattices, then* $\alpha$ *is continuous, i.e.,* $\forall F \subseteq X.\, \alpha(\bigsqcup F) = \bigsqcup \alpha(F)$.

If two property mappings, $\tau$ and $\sigma$, form a Galois connection on trace properties ordered by set inclusion, Lemma 2.3 (with $\alpha = \tau$ and $\gamma = \sigma$) tells us that they satisfy the ideal conditions we discussed above, i.e., $\tau(\sigma(\pi_{\mathbf{T}})) \subseteq \pi_{\mathbf{T}}$ and $\sigma(\tau(\pi_{\mathsf{S}})) \supseteq \pi_{\mathsf{S}}$.[4]

The two ideal conditions on $\tau$ and $\sigma$ are sufficient to show the equivalence of the criteria they define, respectively $\mathsf{TP}^{\tau}$ and $\mathsf{TP}^{\sigma}$.

**Theorem 2.4 ($\mathsf{TP}^{\tau}$ and $\mathsf{TP}^{\sigma}$ coincide 🐓).** *Let* $\tau : 2^{\mathsf{Trace_S}} \rightleftarrows 2^{\mathbf{Trace_T}} : \sigma$ *be a Galois connection, with* $\tau$ *and* $\sigma$ *the lower and upper adjoints (resp.). Then* $\mathsf{TP}^{\tau} \iff \mathsf{TP}^{\sigma}$.

#### 2.2 Trace Relations and Property Mappings

We now investigate the relation between $\mathsf{CC}^{\sim}$, $\mathsf{TP}^{\tau}$ and $\mathsf{TP}^{\sigma}$. We show that for a trace relation and its corresponding Galois connection (Lemma 2.7), the three criteria are equivalent (Theorem 2.8). This equivalence offers interesting insights for both verification and design of a correct compiler. For a $\mathsf{CC}^{\sim}$ compiler, the equivalence makes explicit both the guarantees one has after compilation ($\tilde{\tau}$) and source proof obligations to ensure the satisfaction of a given target property ($\tilde{\sigma}$). On the other hand, a compiler designer might first determine the target guarantees the compiler itself must provide, i.e., $\tau$, and then prove an equivalent statement, $\mathsf{CC}^{\sim}$, for which more convenient proof techniques exist in the literature [7, 58].

**Definition 2.5 (Existential and Universal Image [20]).** *Given any two sets* $X$ *and* $Y$ *and a relation* $\sim \subseteq A \times B$*, define its existential or direct image,* $\tilde{\tau} : 2^X \to 2^Y$ *and its universal image,* $\tilde{\sigma} : 2^Y \to 2^X$ *as follows:*

$$\tilde{\tau} = \lambda\, \pi \in 2^X.\ \{y \mid \exists x.\, x \sim y \wedge x \in \pi\}\,; \tilde{\sigma} = \lambda\, \pi \in 2^Y.\ \{x \mid \forall y.\, x \sim y \Rightarrow y \in \pi\}\,.$$

When trace relations are considered, the existential and universal images can be used to instantiate Definition 2.1 leading to the trinitarian view already mentioned in §1.

**Theorem 2.6 (Trinitarian View 🐓).** *For any trace relation* $\sim$ *and its existential and universal images* $\tilde{\tau}$ *and* $\tilde{\sigma}$*, we have:* $\mathsf{TP}^{\tilde{\tau}} \iff \mathsf{CC}^{\sim} \iff \mathsf{TP}^{\tilde{\sigma}}$.

This result relies both on Theorem 2.4 and on the fact that the existential and universal images of a trace relation form a Galois connection (🐓). Below we further generalize this result (Theorem 2.8) relying on a bijective correspondence between trace relations and Galois connections on properties.

**Lemma 2.7 (Trace relations ≅ Galois connections on trace properties).** *The function* $\sim \mapsto \tilde{\tau} \leftrightarrows \tilde{\sigma}$ *that maps a trace relation to its existential and universal images is a bijection between trace relations* $2^{\mathsf{Trace_S} \times \mathbf{Trace_T}}$ *and Galois connections on trace properties* $2^{\mathsf{Trace_S}} \leftrightarrows 2^{\mathbf{Trace_T}}$*. Its inverse is* $\tau \leftrightarrows \sigma \mapsto \hat{\sim}$*, where* $\mathsf{s} \mathrel{\hat{\sim}} \mathbf{t} \equiv \mathbf{t} \in \tau(\{\mathsf{s}\})$.

[4] While target traces are often *"more concrete"* than source ones, trace properties $2^{\mathrm{Trace}}$ (which in Coq we represent as the function type Trace→Prop) are contravariant in Trace and thus target properties correspond to the *abstract domain*.

*Proof.* Gardiner et al. [20] show that the existential image is a functor from the category of sets and relations to the category of predicate transformers, mapping a set $X \mapsto 2^X$ and a relation $\sim \subseteq X \times Y \mapsto \tilde{\tau} : 2^X \to 2^Y$. They also show that such a functor is an isomorphism – hence bijective – when one considers only monotonic predicate transformers that have a – unique – upper adjoint. The universal image of $\sim$, $\tilde{\sigma}$, is the unique adjoint of $\tilde{\tau}$ (🐓), hence $\sim \mapsto \tilde{\tau} \leftrightarrows \tilde{\sigma}$ is itself bijective. □

The bijection just introduced allows us to generalize Theorem 2.6 and switch between the three views of compiler correctness described earlier at will.

**Theorem 2.8 (Correspondence of Criteria).** *For any trace relation $\sim$ and corresponding Galois connection $\tau \leftrightarrows \sigma$, we have:* $\mathsf{TP}^{\tau} \iff \mathsf{CC}^{\sim} \iff \mathsf{TP}^{\sigma}$.

*Proof.* For a trace relation $\sim$ and the Galois connection $\tilde{\tau} \leftrightarrows \tilde{\sigma}$, the result follows from Theorem 2.6. For a Galois connection $\tau \leftrightarrows \sigma$ and $\hat{\sim}$, use Lemma 2.7 to conclude that the existential and universal images of $\hat{\sim}$ coincide with $\tau$ and $\sigma$, respectively; the goal then follows from Theorem 2.6. □

We conclude by explicitly noting that sometimes the lifted properties may be trivial: the target guarantee can be the true property (the set of all traces), or the source obligation the false property (the empty set of traces). This might be the case when source observations abstract away too much information (§3.2 presents an example).

#### 2.3 Preservation of Subset-Closed Hyperproperties

A $\mathsf{CC}^{=}$ compiler ensures the preservation not only of trace properties, but also of all subset-closed hyperproperties, which are known to be preserved by refinement [14]. An example of a subset-closed hyperproperty is *noninterference* [14]; a $\mathsf{CC}^{=}$ compiler thus guarantees that if W is noninterfering with respect to the inputs and outputs in the trace then so is W↓. To be able to talk about how (hyper)properties such as noninterference are preserved, in this section we propose another trinitarian view involving $\mathsf{CC}^{\sim}$ and preservation of subset-closed hyperproperties (Theorem 2.11), slightly weakened in that source and target property mappings will need to be closed under subsets.

First, recall that a program satisfies a hyperproperty when its complete set of traces, which from now on we will call its *behavior*, is a member of the hyperproperty [14].

**Definition 2.9 (Hyperproperty Satisfaction).** *A program $W$ satisfies a hyperproperty $H$, written $W \models H$, iff* $beh(W) \in H$, *where* $beh(W) = \{t \mid W \rightsquigarrow t\}$.

Hyperproperty preservation is a strong requirement in general. Fortunately, many interesting hyperproperties are *subset-closed* ($SCH$ for short), which simplifies their preservation since it suffices to show that the behaviors of the compiled program refine the behaviors of the source one, which coincides with the statement of $\mathsf{CC}^{=}$.

To talk about hyperproperty preservation in the trace-relating setting, we need an interpretation of source hyperproperties into the target and vice versa. The one we consider builds on top of the two trace property mappings $\tau$ and $\sigma$, which are naturally lifted to hyperproperty mappings. This way we are able to extract two hyperproperty mappings from a trace relation similarly to §2.2:

**Definition 2.10 (Lifting property mappings to hyperproperty mappings).** *Let* $\tau : 2^{\mathsf{Trace_S}} \to 2^{\mathbf{Trace_T}}$ *and* $\sigma : 2^{\mathbf{Trace_T}} \to 2^{\mathsf{Trace_S}}$ *be arbitrary property mappings. The images of* $\mathsf{H_S} \in 2^{2^{\mathsf{Trace_S}}}$, $\mathbf{H_T} \in 2^{2^{\mathbf{Trace_T}}}$ *under* $\tau$ *and* $\sigma$ *are, respectively:*

$$\tau(\mathsf{H_S}) = \{\tau(\pi_\mathsf{S}) \mid \pi_\mathsf{S} \in \mathsf{H_S}\}; \qquad \sigma(\mathbf{H_T}) = \{\sigma(\boldsymbol{\pi}_\mathbf{T}) \mid \boldsymbol{\pi}_\mathbf{T} \in \mathbf{H_T}\}.$$

Formally we are defining two new mappings, this time on hyperproperties, but by a small abuse of notation we still denote them by $\tau$ and $\sigma$.

Interestingly, it is not possible to apply the argument used for $\mathsf{CC}^=$ to show that a $\mathsf{CC}^\sim$ compiler guarantees $\mathsf{W}{\downarrow} \models \tilde{\tau}(\mathsf{H_S})$ whenever $\mathsf{W} \models \mathsf{H_S}$. This is in fact not true because direct images do not necessarily preserve subset-closure [36, 44]. To fix this we close the image of $\tilde{\tau}$ and $\tilde{\sigma}$ under subsets (denoted as $Cl_\subseteq$) and obtain:

**Theorem 2.11 (Preservation of Subset-Closed Hyperproperties 🐓).** *For any trace relation* $\sim$ *and its existential and universal images lifted to hyperproperties,* $\tilde{\tau}$ *and* $\tilde{\sigma}$*, and for* $Cl_\subseteq(H) = \{\pi \mid \exists \pi' \in H.\, \pi \subseteq \pi'\}$*, we have:*

$$\mathsf{SCHP}^{Cl_\subseteq \circ \tilde{\tau}} \iff \mathsf{CC}^\sim \iff \mathsf{SCHP}^{Cl_\subseteq \circ \tilde{\sigma}}, \textit{ where}$$

$$\mathsf{SCHP}^{Cl_\subseteq \circ \tilde{\tau}} \equiv \forall \mathsf{W} \forall \mathsf{H_S} \in \mathsf{SCH_S}. \mathsf{W} \models \mathsf{H_S} \Rightarrow \mathsf{W}{\downarrow} \models Cl_\subseteq(\tilde{\tau}(\mathsf{H_S}));$$

$$\mathsf{SCHP}^{Cl_\subseteq \circ \tilde{\sigma}} \equiv \forall \mathsf{W} \forall \mathbf{H_T} \in \mathbf{SCH_T}. \mathsf{W} \models Cl_\subseteq(\tilde{\sigma}(\mathbf{H_T})) \Rightarrow \mathsf{W}{\downarrow} \models \mathbf{H_T}.$$

Theorem 2.11 makes us aware of the potential loss of precision when interested in preserving subset-closed hyperproperties through compilation. In §4 we focus on a security relevant subset-closed hyperproperty, noninterference, and show that such a loss of precision can be intended as a declassification of noninterference.

## 3 Instances of Trace-Relating Compiler Correctness

The trace-relating view of compiler correctness above can serve as a unifying framework for studying a range of interesting compilers. This section provides several representative instantiations of the framework: source languages with undefined behavior that compilation can turn into arbitrary target behavior (§3.1), target languages with resource exhaustion that cannot happen in the source (§3.2), changes in the representation of values (§3.3), and differences in the granularity of data and observable events (§3.4).

### 3.1 Undefined Behavior

We start by expanding upon the discussion of undefined behavior in §1. We first study the model of CompCert, where source and target alphabets are the same, including the event for undefined behavior. The trace relation weakens equality by allowing undefined behavior to be replaced with an arbitrary sequence of events.

*Example 3.1 (CompCert-like Undefined Behavior Relation).* Source and target traces are sequences of events drawn from $\Sigma$, where $Goes\_wrong \in \Sigma$ is a terminal event that represents an undefined behavior. We then use the trace relation from the introduction:

$$\mathsf{s} \sim \mathsf{t} \quad \equiv \quad \mathsf{s} = \mathsf{t} \vee \exists m \leq \mathsf{t}.\ \mathsf{s} = m \cdot Goes\_wrong.$$

Each trace of a target program produced by a $\mathsf{CC}^\sim$ compiler is either also a trace of the original source program or it has a finite prefix that the source program also produces, immediately before encountering undefined behavior. As explained in §1, one of the correctness theorems in CompCert can be rephrased as this variant of $\mathsf{CC}^\sim$.

We proved that the property mappings induced by the relation can be written as (🐓):

$$\tilde{\sigma}(\pi_T) = \{s \mid s \in \pi_T \wedge s \neq m \cdot Goes\_wrong\} \cup \{m \cdot Goes\_wrong \mid \forall t.\ m \leq t \implies t \in \pi_T\}\,;$$
$$\tilde{\tau}(\pi_S) = \{t \mid t \in \pi_S\} \cup \{t \mid \exists m \leq t.\ m \cdot Goes\_wrong \in \pi_S\}\,.$$

These two mappings explain what a $CC^{\sim}$ compiler ensures for the $\sim$ relation above. The target-to-source mapping $\tilde{\sigma}$ states that to prove that a compiled program has a property $\pi_T$ using source-level reasoning, one has to prove that any trace produced by the source program must either be a target trace satisfying $\pi_T$ or have undefined behavior, but only provided that *any continuation* of the trace substituted for the undefined behavior satisfies $\pi_T$. The source-to-target mapping $\tilde{\tau}$ states that by compiling a program satisfying a property $\pi_S$ we obtain a program that produces traces that satisfy the same property or that extend a source trace that ends in undefined behavior.

These definitions can help us reason about programs. For instance, $\tilde{\sigma}$ specifies that, to prove that an event does not happen in the target, it is not enough to prove that it does not happen in the source: it is also necessary to prove that the source program is does not have any undefined behavior (second disjunct). Indeed, if it had an undefined behavior, its continuations could exhibit the unwanted event. ⊡

This relation can be easily generalized to other settings. For instance, consider the setting in which we compile down to a low-level language like machine code. Target traces can now contain new events that cannot occur in the source: indeed, in modern architectures like x86 a compiler typically uses only a fraction of the available instruction set. Some instructions might even perform dangerous operations, such as writing to the hard drive. Formally, the source and target do not have the same events any more. Thus, we consider a source alphabet $\Sigma_S = \Sigma \cup \{Goes\_wrong\}$, and a target alphabet $\Sigma_T = \Sigma \cup \Sigma'$. The trace relation is defined in the same way and we obtain the same property mappings as above, except that since target traces now have more events (some of which may be dangerous), and the arbitrary continuations of target traces get more interesting. For instance, consider a new event that represents writing data on the hard drive, and suppose we want to prove that this event cannot happen for a compiled program. Then, proving this property requires exactly proving that the source program exhibits no undefined behavior [11]. More generally, what one can prove about target-only events can only be either that they cannot appear (because there is no undefined behavior) or that any of them can appear (in the case of undefined behavior).

In §5.2 we study a similar example, showing that even in a safe language linked adversarial contexts can cause dangerous target events that have no source correspondent.

#### 3.2 Resource Exhaustion

Let us return to the discussion about resource exhaustion in §1.

*Example 3.2 (Resource Exhaustion).* We consider traces made of events drawn from $\Sigma_S$ in the source, and $\Sigma_T = \Sigma_S \cup \{\mathbf{Resource\_Limit\_Hit}\}$ in the target. Recall the trace relation for resource exhaustion:

$$s \sim t \quad \equiv \quad s = t \vee \exists m \leq s.\ t = m \cdot \mathbf{Resource\_Limit\_Hit}.$$

Formally, this relation is similar to the one for undefined behavior, except this time it is the target trace that is allowed to end early instead of the source trace.

The induced trace property mappings $\tilde{\sigma}$ and $\tilde{\tau}$ are the following:

$$\tilde{\sigma}(\pi_T) = \{\mathsf{s} \mid \mathsf{s} \in \pi_T\} \cap \{\mathsf{s} \mid \forall m \leq \mathsf{s}.\ m \cdot \mathbf{Resource\_Limit\_Hit} \in \pi_T\};$$

$$\tilde{\tau}(\pi_S) = \pi_S \cup \{m \cdot \mathbf{Resource\_Limit\_Hit} \mid \exists \mathsf{s} \in \pi_S.\ m \leq \mathsf{s}\}.$$

These capture the following intuitions. The target-to-source mapping $\tilde{\sigma}$ states that to prove a property of the compiled program one has to show that the traces of the source program satisfy two conditions: (1) they must also satisfy the target property; and (2) the termination of every one of their prefixes by a resource exhaustion error must be allowed by the target property. This is rather restrictive: any property that prevents resource exhaustion cannot be proved using source-level reasoning. Indeed, if $\pi_T$ does not allow resource exhaustion, then $\tilde{\sigma}(\pi_T) = \varnothing$. This is to be expected since resource exhaustion is simply not accounted for at the source level. The other mapping $\tilde{\tau}$ states that a compiled program produces traces that either belong to the same properties as the traces of the source program or end early due to resource exhaustion.

In this example, safety properties [31] are mapped (in both directions) to other safety properties. This can be desirable for a relation: since safety properties are usually easier to reason about, one interested only in safety properties at the target can reason about them using source-level reasoning tools for safety properties.

The compiler correctness theorem in CakeML is an instance of $\mathsf{CC}^{\sim}$ for the $\sim$ relation above. We have also implemented two small compilers that are correct for this relation. The full details can be found in the Coq development in the supplementary materials. The first compiler goes from a simple expression language (similar to the one in §3.3 but without inputs) to the same language except that execution is bounded by some amount of fuel: each execution step consumes some amount of fuel and execution immediately halts when it runs out of fuel. The compiler is the identity.

The second compiler is more interesting: we proved this $\mathsf{CC}^{\sim}$ instance for a variant of a compiler from a WHILE language to a simple stack machine by Xavier Leroy [35]. We enriched the two languages with outputs and modified the semantics of the stack machine so that it falls into an error state if the stack reaches a certain size. The proof uses a standard forward simulation modified to account for failure. ⊡

We conclude this subsection by noting that the resource exhaustion relation and the undefined behavior relation from the previous subsection can easily be combined. Indeed, given a relation $\sim_{\text{UB}}$ and a relation $\sim_{\text{RE}}$ defined as above on the same sets of traces, we can build a new relation $\sim$ that allows both refinement of undefined behavior and resource exhaustion by taking their union: $\mathsf{s} \sim \mathsf{t} \equiv \mathsf{s} \sim_{\text{UB}} \mathsf{t} \vee \mathsf{s} \sim_{\text{RE}} \mathsf{t}$. A compiler that is $\mathsf{CC}^{\sim_{\text{UB}}}$ or $\mathsf{CC}^{\sim_{\text{RE}}}$ is trivially $\mathsf{CC}^{\sim}$, though the converse is not true.

#### 3.3 Different Source and Target Values

We now illustrate trace-relating compilation for a translation mapping source-level booleans to target-level natural numbers. Given the simplicity of this compiler, most of the details of the formalization are deferred to the online appendix.

The source language is a pure, statically typed expression language whose expressions $\mathsf{e}$ include naturals $\mathsf{n}$, booleans $\mathsf{b}$, conditionals, arithmetic and relational operations, boolean inputs $\mathsf{in_b}$ and natural inputs $\mathsf{in_n}$. A trace $\mathsf{s}$ is a list of inputs $\mathsf{is}$ paired with a result $\mathsf{r}$, which can be a natural, a boolean, or an error. Well-typed programs never produce error. Types $\mathsf{ty}$ are either $\mathsf{N}$ (naturals) or $\mathsf{B}$ (booleans); typing is standard. The source language has a standard big-step operational semantics ($\mathsf{e} \rightsquigarrow \langle \mathsf{is}, \mathsf{r} \rangle$) which tells how an expression $\mathsf{e}$ generates a trace $\langle \mathsf{is}, \mathsf{r} \rangle$. The target language is analogous, except that it is untyped, only has naturals $\mathbf{n}$ and its only inputs are naturals $\mathbf{in_n}$. The semantics of the target language is also given in big-step style. Since we only have naturals and all expressions operate on them, no error result is possible in the target.

The compiler is homomorphic, translating a source expression to the same target expression; the only differences are natural numbers (and conditionals), as noted below.

$$\mathsf{true}{\downarrow} = \mathbf{1} \quad \mathsf{in_b}{\downarrow} = \mathbf{in_n} \qquad \mathsf{e_1} \leq \mathsf{e_2}{\downarrow} = \mathbf{if}\ \mathsf{e_1}{\downarrow} \leq \mathsf{e_2}{\downarrow}\ \mathbf{then\ 1\ else\ 0}$$

$$\mathsf{false}{\downarrow} = \mathbf{0} \quad \mathsf{in_n}{\downarrow} = \mathbf{in_n} \quad \mathsf{if}\ \mathsf{e_1}\ \mathsf{then}\ \mathsf{e_2}\ \mathsf{else}\ \mathsf{e_3}{\downarrow} = \mathbf{if}\ \mathsf{e_1}{\downarrow} \leq \mathbf{0}\ \mathbf{then}\ \mathsf{e_3}{\downarrow}\ \mathbf{else}\ \mathsf{e_2}{\downarrow}$$

When compiling an *if-then-else* the target condition $\mathsf{e_1}{\downarrow} \leq \mathbf{0}$ is used to check that $\mathsf{e_1}$ is false, and therefore the *then* and *else* branches of the source are swapped in the target.

**Relating Traces.** We relate basic values (naturals and booleans) in a non-injective fashion as noted below. Then, we extend the relation to lists of inputs pointwise (Rules Empty and Cons) and lift that relation to traces (Rules Nat and Bool).

$$\mathsf{n} \sim \mathbf{n} \qquad \mathsf{true} \sim \mathbf{n} \quad \text{if } \mathbf{n} > 0 \qquad \mathsf{false} \sim \mathbf{0}$$

$$\text{(Empty)}\ \frac{}{\varnothing \sim \boldsymbol{\varnothing}} \qquad \text{(Cons)}\ \frac{\mathsf{i} \sim \mathbf{i} \quad \mathsf{is} \sim \mathbf{is}}{\mathsf{i} \cdot \mathsf{is} \sim \mathbf{i} \cdot \mathbf{is}} \qquad \text{(Nat)}\ \frac{\mathsf{is} \sim \mathbf{is} \quad \mathsf{n} \sim \mathbf{n}}{\langle \mathsf{is}, \mathsf{n} \rangle \sim \langle \mathbf{is}, \mathbf{n} \rangle} \qquad \text{(Bool)}\ \frac{\mathsf{is} \sim \mathbf{is} \quad \mathsf{b} \sim \mathbf{n}}{\langle \mathsf{is}, \mathsf{b} \rangle \sim \langle \mathbf{is}, \mathbf{n} \rangle}$$

**Property mappings.** The property mappings $\tilde{\sigma}$ and $\tilde{\tau}$ induced by the trace relation $\sim$ defined above capture the intuition behind encoding booleans as naturals:

- the source-to-target mapping allows $\mathsf{true}$ to be encoded by any non-zero number;
- the target-to-source mapping requires that $\mathbf{0}$ be replaceable by *both* $\mathsf{0}$ and $\mathsf{false}$.

**Compiler correctness.** With the relation above, the compiler is proven to satisfy $\mathsf{CC}^{\sim}$.

**Theorem 3.3** **($\cdot{\downarrow}$ is correct 🐓).** $\cdot{\downarrow}$ *is* $\mathsf{CC}^{\sim}$.

**Simulations with different traces.** The difficulty in proving Theorem 3.3 arises from the trace-relating compilation setting: For compilation chains that have the same source and target traces, it is customary to prove compiler correctness using a forward simulation (i.e., a simulation between source and target transition system); then, using determinacy [18, 39] of the target language and input totality [19, 63] (aka receptiveness) of the source, this forward simulation is flipped into a backward simulation (a simulation between target and source transition system), as described by Beringer et al. [7], Leroy [34]. This flipping is useful because forward simulations are often much easier to prove (by induction on the transitions of the source) than backward ones, as it is the case here.

We first give the main idea of the flipping proof, when the inputs are the same in the source and the target [7, 34]. We only consider inputs, as it is the most interesting case, since with determinacy, nondeterminism only occurs on inputs. Given a forward simulation $\mathcal{R}$, and a target program $\mathbf{W_T}$ that simulates a source program $\mathsf{W_S}$, $\mathbf{W_T}$ is able to perform an input iff so is $\mathsf{W_S}$: otherwise, say for instance that $\mathsf{W_S}$ performs an output, by forward simulation $\mathbf{W_T}$ would also perform an output, which is impossible because of determinacy. By input totality of the source, $\mathsf{W_S}$ must be able to perform the exact same input as $\mathbf{W_T}$; using forward simulation and determinacy, the resulting programs must be related.

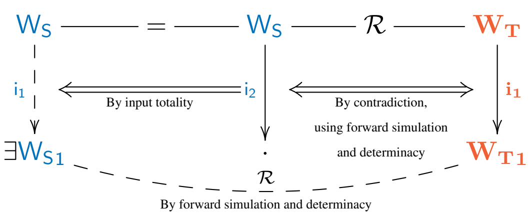

However, our trace relation is not injective (both $0$ and $\mathsf{false}$ are mapped to $\mathbf{0}$), therefore these arguments do not apply: not all possible inputs of target programs are accounted for in the forward simulation. We thus have to strengthen the forward simulation assumption, requiring the following additional property to hold, for any source program $\mathsf{W_S}$ and target program $\mathbf{W_T}$ related by the forward simulation $\mathcal{R}$.

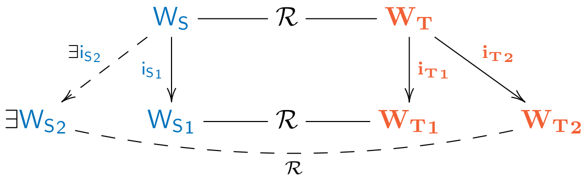

where $\mathsf{i_{S1}} \sim \mathbf{i_{T1}}$
$\mathsf{i_{S1}} \sim \mathbf{i_{T2}}$
$\mathsf{i_{S2}} \sim \mathbf{i_{T2}}$

We say that a forward simulation for which this property holds is *flippable*. For our example compiler, a flippable forward simulation works as follows: whenever a boolean input occurs in the source, the target program must perform every strictly positive input $\mathbf{n}$ (and not just $\mathbf{1}$, as suggested by the compiler). Using this property, determinacy of the target, input totality of the source, as well as the fact that any target input has an inverse image through the relation, we can indeed show that the forward simulation can be turned into a backward one: starting from $\mathsf{W_S}\ \mathcal{R}\ \mathbf{W_T}$ and an input $\mathbf{i_{T2}}$, we show that there is $\mathsf{i_{S1}}$ and $\mathbf{i_{T2}}$ as in the diagram above, using the same arguments as when the inputs are the same; because the simulation is flippable, we can close the diagram, and obtain the existence of an adequate $\mathsf{i_{S2}}$. From this we obtain $\mathsf{CC}^{\sim}$.

In fact, we have proven a completely general 'flipping theorem', with this flippable hypothesis on the forward simulation (🐓). We have also shown that if the relation $\sim$ defines a bijection between the inputs of the source and the target, then any forward simulation is flippable, hence reobtaining the usual proof technique [7, 34] as a special case. This flipping theorem is further discussed in the online appendix.

#### 3.4 Abstraction Mismatches

We now consider how to relate traces where a single source action is compiled to multiple target ones. To illustrate this, we take a pure, statically-typed source language that can output (nested) pairs of arbitrary size, and a pure, *untyped* target language where sent values have a fixed size. Concretely, the source is analogous to the language of §3.3, except that it does not have inputs or booleans and it has an expression $\mathsf{send\ e}$, which can emit a (nested) pair $\mathsf{e}$ of values in a single action. That is, given that $\mathsf{e}$ reduces to a pair, e.g., $\langle \mathsf{v1}, \langle \mathsf{v2}, \mathsf{v3}\rangle\rangle$, expression $\mathsf{send}\ \langle \mathsf{v1}, \langle \mathsf{v2}, \mathsf{v3}\rangle\rangle$ emits action $\langle \mathsf{v1}, \langle \mathsf{v2}, \mathsf{v3}\rangle\rangle$. That expression is compiled into a sequence of individual sends in the target language $\mathbf{send\ v1}\ ;\ \mathbf{send\ v2}\ ;\ \mathbf{send\ v3}$, since in the target, $\mathbf{send\ e}$ sends the value that $\mathbf{e}$ reduces to, but the language has no pairs.

Due to space constraints we omit the full formalization of these simple languages and of the homomorphic compiler ($(\cdot)\downarrow : \mathsf{e} \to \mathbf{e}$). The only interesting bit is the compilation of the $\mathsf{send}\ \cdot$ expression, which relies on the gensend $(\cdot)$ function below. That function takes a source expression of a given type and returns a sequence of target $\mathbf{send}\ \cdot$ instructions that send each element of the expression.

$$\texttt{gensend}\,(\vdash \mathsf{e} : \tau) = \begin{cases} \mathbf{send}\ (\vdash \mathsf{e} : \mathsf{N})\downarrow & \text{if } \tau = \mathsf{N} \\ \texttt{gensend}\,(\vdash \mathsf{e.1} : \tau'); \texttt{gensend}\,(\vdash \mathsf{e.2} : \tau'') & \text{if } \tau = \tau' \times \tau'' \end{cases}$$

**Relating Traces.** We start with the trivial relation between numbers: $\mathsf{n} \sim^0 \mathbf{n}$, i.e., numbers are related when they are the same. We cannot build a relation between single actions since a single source action is related to multiple target ones. Therefore, we define a relation between a source action $\mathsf{M}$ and a target trace $\mathbf{t}$ (a list of numbers), inductively on the structure of $\mathsf{M}$ (which is a pair of values, and values are natural numbers or pairs).

$$\frac{\mathsf{n} \sim^0 \mathbf{n} \qquad \mathsf{n}' \sim^0 \mathbf{n}'}{\langle \mathsf{n}, \mathsf{n}' \rangle \sim \mathbf{n} \cdot \mathbf{n}'}\ (\text{Trace-Rel-N-N}) \qquad \frac{\mathsf{n} \sim^0 \mathbf{n} \qquad \mathsf{M} \sim \mathbf{t}}{\langle \mathsf{n}, \mathsf{M} \rangle \sim \mathbf{n} \cdot \mathbf{t}}\ (\text{Trace-Rel-N-M})$$

$$\frac{\mathsf{M} \sim \mathbf{t} \qquad \mathsf{n} \sim^0 \mathbf{n}}{\langle \mathsf{M}, \mathsf{n} \rangle \sim \mathbf{t} \cdot \mathbf{n}}\ (\text{Trace-Rel-M-N}) \qquad \frac{\mathsf{M} \sim \mathbf{t} \qquad \mathsf{M}' \sim \mathbf{t}'}{\langle \mathsf{M}, \mathsf{M}' \rangle \sim \mathbf{t} \cdot \mathbf{t}'}\ (\text{Trace-Rel-M-M})$$

A pair of naturals is related to the two actions that send each element of the pair (Rule Trace-Rel-N-N). If a pair is made of sub-pairs, we require all such sub-pairs to be related (Rules Trace-Rel-N-M to Trace-Rel-M-M). We build on these rules to define the $\mathsf{s} \sim \mathbf{t}$ relation between source and target traces for which the compiler is correct (Theorem 3.4). Trivially, traces are related when they are both empty. Alternatively, given related traces, we can concatenate a source action and a second target trace provided that they are related (Rule Trace-Rel-Single).

$$\frac{\mathsf{s} \sim \mathbf{t} \qquad \mathsf{M} \sim \mathbf{t}'}{\mathsf{s} \cdot \mathsf{M} \sim \mathbf{t} \cdot \mathbf{t}'}\ (\text{Trace-Rel-Single})$$

**Theorem 3.4** **($(\cdot)\downarrow$ is correct).** $(\cdot)\downarrow$ *is* $\mathsf{CC}^{\sim}$.

With our trace relation, the trace property mappings capture the following intuitions:

- The target-to-source mapping states that a source property can reconstruct target action as it sees fit. For example, trace $\mathbf{4} \cdot \mathbf{6} \cdot \mathbf{5} \cdot \mathbf{7}$ is related to $\langle 4, 6 \rangle \cdot \langle 5, 7 \rangle$ and $\langle\langle 4, \langle 6, \langle 5, 7 \rangle\rangle\rangle\rangle$ (and many more variations). This gives freedom to the source implementation of a target behavior, which follows from the non-injectivity of $\sim$.[5]
- The source-to-target mapping "forgets" about the way pairs are nested, but is faithful w.r.t. the values $\mathsf{v_i}$ contained in a message. Notice that source safety properties are always mapped to target safety properties. For instance, if $\pi_\mathsf{S} \in \mathsf{Safety_S}$ prescribes that some bad number is never sent, then $\tilde{\tau}(\pi_\mathsf{S})$ prescribes the same number is never sent in the target and $\tilde{\tau}(\pi_\mathsf{S}) \in \mathbf{Safety_T}$. Of course if $\pi_\mathsf{S} \in \mathsf{Safety_S}$ prescribes that a particular nested pairing like $\langle 4, \langle 6, \langle 5, 7 \rangle\rangle\rangle$ never happens, then $\tilde{\tau}(\pi_\mathsf{S})$ is still a target safety property, but the trivial one, since $\tilde{\tau}(\pi_\mathsf{S}) = \top \in \mathbf{Safety_T}$.

### 4 Trace-Relating Compilation and Noninterference Preservation

When source and target observations are drawn from the same set, a correct compiler ($\mathsf{CC}^{=}$) is enough to ensure the preservation of all subset-closed hyperproperties, in particular of *noninterference* (NI) [22], as also mentioned at the beginning of §2.3. In the

[5] Making $\sim$ injective is a matter of adding open and close parenthesis actions in target traces.

scenario where target observations are strictly more informative than source observations, the best guarantee one may expect from a correct trace-relating compiler ($\mathsf{CC}^{\sim}$) is a *weakening* (or *declassification*) of target noninterference that matches the noninterference property satisfied in the source. To formalize this reasoning, this section applies the trinitarian view of trace-relating compilation to the general framework of abstract noninterference (ANI) [21].

We first define NI and explain the issue of preserving source NI via a $\mathsf{CC}^{\sim}$ compiler. We then introduce ANI, which allows characterizations of various forms of noninterference, and formulate a general theory of ANI preservation via $\mathsf{CC}^{\sim}$. We also study how to deal with cases such as undefined behavior in the target. Finally, we answer the dual question, i.e., which source NI should be satisfied to guarantee that compiled programs are noninterfering with respect to target observers.

Intuitively, NI requires that publicly observable outputs do not reveal information about private inputs. To define this formally, we need a few additions to our setup. We indicate the (disjoint) *input* and *output* projections of a trace $t$ as $t^{\circ}$ and $t^{\bullet}$ respectively[6]. Denote with $[t]_{low}$ the equivalence class of a trace $t$, obtained using a standard low-equivalence relation that relates low (public) events only if they are equal, and ingores any difference between private events. Then, NI for source traces can be defined as:

$$\mathsf{NI_S} = \{\pi_\mathsf{S} \mid \forall \mathsf{s_1 s_2} \in \pi_\mathsf{S}.\ [\mathsf{s}_1^{\circ}]_{low} = [\mathsf{s}_2^{\circ}]_{low} \Rightarrow [\mathsf{s}_1^{\bullet}]_{low} = [\mathsf{s}_2^{\bullet}]_{low}\ \}\,.$$

That is, source NI comprises the sets of traces that have equivalent low output projections as long as their low input projections are equivalent.

**Trace-Relating Compilation and Noninterference.** When additional observations are possible in the target, it is unclear whether a noninterfering source program is compiled to a noninterfering target program or not, and if so, whether the notion of NI in the target is the expected or desired one. We illustrate this issue considering a scenario where target traces extend source ones by exposing the execution time. While source noninterference $\mathsf{NI_S}$ requires that private inputs do not affect public outputs, $\mathbf{NI_T}$ additionally requires that the execution time is not affected by private inputs.

To model the scenario described, let $\mathsf{Trace_S}$ denote the set of traces in the source, and $\mathbf{Trace_T} = \mathsf{Trace_S} \times \mathbb{N}^{\omega}$ be the set of target traces, where $\mathbb{N}^{\omega} \triangleq \mathbb{N} \cup \{\omega\}$. Target traces have two components: a source trace, and a natural number that denotes the time spent to produce the trace ($\omega$ if infinite). Notice that if two source traces $\mathsf{s_1}, \mathsf{s_2}$, are low-equivalent then $\{\mathsf{s_1}, \mathsf{s_2}\} \in \mathsf{NI_S}$ and $\{(\mathsf{s_1}, \mathbf{42}), (\mathsf{s_1}, \mathbf{42})\} \in \mathbf{NI_T}$, but $\{(\mathsf{s_1}, \mathbf{42}), (\mathsf{s_2}, \mathbf{43})\} \notin \mathbf{NI_T}$ and $\{(\mathsf{s_1}, \mathbf{42}), (\mathsf{s_2}, \mathbf{42}), (\mathsf{s_1}, \mathbf{43}), (\mathsf{s_2}, \mathbf{43})\} \notin \mathbf{NI_T}$.

Consider the following straightforward trace relation, which relates a source trace to any target trace whose first component is equal to it, irrespective of execution time:

$$\mathsf{s} \sim \mathbf{t} \quad \equiv \quad \exists \mathbf{n}.\ \mathbf{t} = (\mathsf{s}, \mathbf{n}).$$

A compiler is $\mathsf{CC}^{\sim}$ if any trace that can be exhibited in the target can be simulated in the source in some amount of time. For such a compiler Theorem 2.11 says that if $\mathsf{W}$ satisfies $\mathsf{NI_S}$, then $\mathsf{W}{\downarrow}$ satisfies $Cl_{\subseteq} \circ \tilde{\tau}(\mathsf{NI_S})$, which however is strictly weaker than $\mathbf{NI_T}$, as it contains, e.g., $\{(\mathsf{s_1}, \mathbf{42}), (\mathsf{s_2}, \mathbf{42}), (\mathsf{s_1}, \mathbf{43}), (\mathsf{s_2}, \mathbf{43})\}$, and one cannot conclude that $\mathsf{W}{\downarrow}$ is noninterfering in the target. It is easy to prove that

[6] Here we only require the projections to be disjoint. Depending on the scenario and the attacker model the projections might record information such as the ordering of events.

$$Cl_{\subseteq} \circ \tilde{\tau}(\mathsf{NI_S}) = Cl_{\subseteq}\left(\{\, \pi_{\mathsf{S}} \times \mathbb{N}^{\omega} \mid \pi_{\mathsf{S}} \in \mathsf{NI_S}\}\right) = \{\, \pi_{\mathsf{S}} \times \mathcal{I} \mid \pi_{\mathsf{S}} \in \mathsf{NI_S} \wedge \mathcal{I} \subseteq \mathbb{N}^{\omega}\},$$

the first equality coming from $\tilde{\tau}(\pi_{\mathsf{S}}) = \pi_{\mathsf{S}} \times \mathbb{N}^{\omega}$, and the second from $\mathsf{NI_S}$ being subset-closed. As we will see, this hyperproperty *can* be characterized as a form of NI, which one might call *timing-insensitive noninterference*, and ensured only against attackers that cannot measure execution time. For this characterization, and to describe different forms of noninterference as well as formally analyze their preservation by a $\mathsf{CC}^{\sim}$ compiler, we rely on the general framework of *abstract noninterference* [21].

**Abstract Noninterference.** ANI [21] is a generalization of NI whose formulation relies on abstractions (in abstract interpretation sense [16]) in order to encompass arbitrary variants of NI. ANI is parameterized by an *observer abstraction* $\rho$, which denotes the distinguishing power of the attacker, and a *selection abstraction* $\phi$, which specifies when to check NI, and therefore captures a form of declassification [54].[7] Formally:

$$ANI^{\rho}_{\phi} = \{\pi \mid \forall t_1 t_2 \in \pi.\ \phi(t_1^{\circ}) = \phi(t_2^{\circ}) \Rightarrow \rho(t_1^{\bullet}) = \rho(t_2^{\bullet})\}.$$

By picking $\phi = \rho = [\cdot]_{low}$, we recover the standard noninterference defined above, where NI must hold for all low inputs (i.e., no declassification of private inputs), and the observational power of the attacker is limited to distinguishing low outputs.

The observational power of the attacker can be weakened by choosing a more liberal relation for $\rho$. For instance, one may limit the attacker to observe the *parity* of output integer values. Another way to weaken ANI is to use $\phi$ to specify that noninterference is only required to hold for a subset of low inputs.

To be formally precise, $\phi$ and $\rho$ are defined over sets of (input and output projections of) traces, so when we write $\phi(t)$ above, this should be understood as a convenience notation for $\phi(\{t\})$. Likewise, $\phi = [\cdot]_{low}$ should be understood as $\phi = \lambda\pi. \bigcup_{t\in\pi}[t]_{low}$, i.e., the powerset lifting of $[\cdot]_{low}$. Additionally, $\phi$ and $\rho$ are required to be upper-closed operators ($uco$)—i.e., monotonic, idempotent and extensive—on the poset that is the powerset of (input and output projections of) traces ordered by inclusion [21].

**Trace-Relating Compilation and ANI for Timing.** We can now reformulate our example with observable execution times in the target in terms of ANI. We have $\mathsf{NI_S} = ANI^{\phi_{\mathsf{S}}}_{\rho_{\mathsf{S}}}$ with $\phi_{\mathsf{S}} = \rho_{\mathsf{S}} = [\cdot]_{low}$. In this case, we can formally describe the hyperproperty that a compiled program $\mathsf{W}\downarrow$ satisfies whenever $\mathsf{W}$ satisfies $\mathsf{NI_S}$ as an instance of ANI:

$$Cl_{\subseteq} \circ \tilde{\tau}(\mathsf{NI_S}) = ANI^{\rho_{\mathbf{T}}}_{\phi_{\mathbf{T}}},$$

$$\text{for } \phi_{\mathbf{T}} = \phi_{\mathsf{S}} \text{ and } \rho_{\mathbf{T}}(\pi_{\mathbf{T}}) = \{(\mathsf{s}, \mathbf{n}) \mid \exists(\mathsf{s}_1, \mathbf{n}_1) \in \pi_{\mathbf{T}}.\ [\mathsf{s}^{\bullet}]_{low} = [\mathsf{s}_1^{\bullet}]_{low}\}.$$

The definition of $\phi_{\mathbf{T}}$ tells us that the trace relation does not affect the selection abstraction. The definition of $\rho_{\mathbf{T}}$ characterizes an observer that cannot distinguish execution times for noninterfering traces (notice that $\mathbf{n}_1$ in the definition of $\rho_{\mathbf{T}}$ is discarded). For instance, $\rho_{\mathbf{T}}(\{(\mathsf{s}, \mathbf{n}_1)\}) = \rho_{\mathbf{T}}(\{(\mathsf{s}, \mathbf{n}_2)\})$, for any $\mathsf{s}$, $\mathbf{n}_1$, $\mathbf{n}_2$. Therefore, in this setting, we know explicitly through $\rho_{\mathbf{T}}$ that a $\mathsf{CC}^{\sim}$ compiler degrades source noninterference to target *timing-insensitive* noninterference.

**Trace-Relating Compilation and ANI in General.** While the particular $\phi_{\mathbf{T}}$ and $\rho_{\mathbf{T}}$ above can be discovered by intuition, we want to know whether there is a systematic way of obtaining them in general. In other words, for *any* trace relation $\sim$ and *any*

[7] ANI includes a third parameter $\eta$, which describes the maximal input variation that the attacker may control. Here we omit $\eta$ (i.e., take it to be the identity) in order to simplify the presentation.

notion of source NI, what property is guaranteed on noninterfering source programs by any $\mathsf{CC}^{\sim}$ compiler?

We can now answer this question generally (Theorem 4.1): any source notion of noninterference expressible as an instance of ANI is mapped to a corresponding instance of ANI in the target, whenever source traces are an abstraction of target ones (i.e., when $\sim$ is a total and surjective map). For this result we consider trace relations that can be split into input and output trace relations (denoted as $\sim \triangleq \langle \overset{\circ}{\sim}, \overset{\bullet}{\sim} \rangle$) such that $\mathsf{s} \sim \mathbf{t} \iff \mathsf{s}^\circ \overset{\circ}{\sim} \mathbf{t}^\circ \wedge \mathsf{s}^\bullet \overset{\bullet}{\sim} \mathbf{t}^\bullet$. The trace relation $\sim$ corresponds to a Galois connection between the sets of trace properties $\tilde{\tau} \leftrightarrows \tilde{\sigma}$ as described in §2.2. Similarly, the pair $\overset{\circ}{\sim}$ and $\overset{\bullet}{\sim}$ corresponds to a pair of Galois connections, $\tilde{\tau}^\circ \leftrightarrows \tilde{\sigma}^\circ$ and $\tilde{\tau}^\bullet \leftrightarrows \tilde{\sigma}^\bullet$, between the sets of input and output properties. In the timing example, time is an output so we have $\sim \triangleq \langle =, \overset{\bullet}{\sim} \rangle$ and $\overset{\bullet}{\sim}$ is defined as $\mathsf{s}^\bullet \overset{\bullet}{\sim} \mathbf{t}^\bullet \equiv \exists \mathbf{n}.\ \mathbf{t}^\bullet = (\mathsf{s}^\bullet, \mathbf{n})$.

**Theorem 4.1 (Compiling ANI).** *Assume traces of source and target languages are related via $\sim \subseteq \mathsf{Trace_S} \times \mathbf{Trace_T}$, $\sim \triangleq \langle \overset{\circ}{\sim}, \overset{\bullet}{\sim} \rangle$ such that $\overset{\circ}{\sim}$ and $\overset{\bullet}{\sim}$ are both total maps from target to source traces, and $\overset{\circ}{\sim}$ is surjective. Assume $\downarrow$ is a $\mathsf{CC}^{\sim}$ compiler, and $\phi_\mathsf{S} \in uco(2^{\mathsf{Trace}^\circ_\mathsf{S}})$, $\rho_\mathsf{S} \in uco(2^{\mathsf{Trace}^\bullet_\mathsf{S}})$.*

*If $\mathsf{W}$ satisfies $ANI^{\rho_\mathsf{S}}_{\phi_\mathsf{S}}$, then $\mathsf{W}\downarrow$ satisfies $ANI^{\rho^\#_\mathbf{T}}_{\phi^\#_\mathbf{T}}$, where $\phi^\#_\mathbf{T}$ and $\rho^\#_\mathbf{T}$ are defined as:*

$$\phi^\#_\mathbf{T} = g^\circ \circ \phi_\mathsf{S} \circ f^\circ; \qquad \rho^\#_\mathbf{T} = g^\bullet \circ \rho_\mathsf{S} \circ f^\bullet \quad \textit{and}$$

$$f^\circ(\pi^\circ_\mathbf{T}) = \left\{ \mathsf{s}^\circ \;\middle|\; \exists \mathbf{t}^\circ \in \pi^\circ_\mathbf{T}.\ \mathsf{s}^\circ \overset{\circ}{\sim} \mathbf{t}^\circ \right\}; \quad g^\circ(\pi^\circ_\mathsf{S}) = \{ \mathbf{t}^\circ \mid \forall \mathsf{s}^\circ.\ \mathsf{s}^\circ \overset{\circ}{\sim} \mathbf{t}^\circ \Rightarrow \mathsf{s}^\circ \in \pi^\circ_\mathsf{S} \}$$

*(and both $f^\bullet$ and $g^\bullet$ are defined analogously).*

For the example above we recover the definitions we justified intuitively, i.e., $\phi^\#_\mathbf{T} = g^\circ \circ \phi_\mathsf{S} \circ f^\circ = \phi_\mathbf{T}$ and $\rho^\#_\mathbf{T} = g^\bullet \circ \rho_\mathsf{S} \circ f^\bullet = \rho_\mathbf{T}$. Moreover, we can prove that if $\overset{\bullet}{\sim}$ also is surjective, $ANI^{\rho^\#_\mathbf{T}}_{\phi^\#_\mathbf{T}} \subseteq Cl_\subseteq \circ \tilde{\tau}(ANI^{\rho_\mathsf{S}}_{\phi_\mathsf{S}})$. Therefore, the derived guarantee $ANI^{\rho^\#_\mathbf{T}}_{\phi^\#_\mathbf{T}}$ is at least as strong as the one that follows by just knowing that the compiler $\downarrow$ is $\mathsf{CC}^{\sim}$.

**Noninterference and Undefined Behavior.** As stated above, Theorem 4.1 does not apply to several scenarios from §3 such as undefined behavior (§3.1), as in those cases the relation $\overset{\bullet}{\sim}$ is not a total map. Nevertheless, we can still exploit our framework to reason about the impact of compilation on noninterference.

Let us consider $\sim \triangleq \langle \overset{\circ}{\sim}, \overset{\bullet}{\sim} \rangle$ where $\overset{\circ}{\sim}$ is any total and surjective map from target to source inputs (e.g., equality) and $\overset{\bullet}{\sim}$ is defined as $\mathsf{s}^\bullet \overset{\bullet}{\sim} \mathbf{t}^\bullet \equiv \mathsf{s}^\bullet = \mathbf{t}^\bullet \vee \exists m^\bullet \le \mathbf{t}^\bullet.\ \mathsf{s}^\bullet = m^\bullet \cdot Goes\_wrong$. Intuitively, a $\mathsf{CC}^{\sim}$ compiler guarantees that no interference can be observed by a target attacker that cannot exploit undefined behavior to learn private information. This intuition can be made formal by the following theorem.

**Theorem 4.2 (Relaxed Compiling ANI).** *Relax the assumptions of Theorem 4.1 by allowing $\overset{\bullet}{\sim}$ to be* any *output trace relation. If $\mathsf{W}$ satisfies $ANI^{\rho_\mathsf{S}}_{\phi_\mathsf{S}}$, then $\mathsf{W}\downarrow$ satisfies $ANI^{\rho^\#_\mathbf{T}}_{\phi^\#_\mathbf{T}}$ where $\phi^\#_\mathbf{T}$ is defined as in Theorem 4.1, and $\rho^\#_\mathbf{T}$ is such that:*

$$\forall \mathsf{s}\, \mathbf{t}.\ \mathsf{s}^\bullet \overset{\bullet}{\sim} \mathbf{t}^\bullet \Rightarrow \ \rho^\#_\mathbf{T}(\mathbf{t}^\bullet) = \rho^\#_\mathbf{T}(\tilde{\tau}^\bullet(\rho_\mathsf{S}(\mathsf{s}^\bullet))).$$

Technically, instead of giving us a *definition* of $\rho^\#_\mathbf{T}$, the theorem gives a *property* of it. The property states that, given a target output trace $\mathbf{t}^\bullet$, the attacker cannot distinguish it from any other target output traces produced by other possible compilations ($\tilde{\tau}^\bullet$) of the source trace $\mathsf{s}$ it relates to, up to the observational power of the source level attacker $\rho_{\mathsf{S}}$. Therefore, given a source attacker $\rho_{\mathsf{S}}$, the theorem characterizes a *family* of attackers that cannot observe any interference for a correctly compiled noninterfering program. Notice that the target attacker $\rho^{\#}_{\mathbf{T}} = \lambda\_.\ \top$ satisfies the premise of the theorem, but defines a trivial hyperproperty, so that we cannot prove in general that $ANI^{\rho^{\#}_{\mathbf{T}}}_{\phi^{\#}_{\mathbf{T}}} \subseteq Cl_{\subseteq} \circ \tilde{\tau}(ANI^{\rho_{\mathsf{S}}}_{\phi_{\mathsf{S}}})$. The same $\rho^{\#}_{\mathbf{T}} = \lambda\_.\ \top$ shows that the family of attackers described in Theorem 4.2 is nonempty, and this ensures the existence of a most powerful attacker among them [21], whose explicit characterization we leave for future work.

**From Target NI to Source NI.** We now explore the dual question: under what hypotheses does trace-relating compiler correctness alone allow target noninterference to be reduced to source noninterference? This is of practical interest, as one would be able to protect from target attackers by ensuring noninterference in the source. This task can be made easier if the source language has some static enforcement mechanism [1, 36].

Let us consider the languages from §3.4 extended with inputting of (pairs of) values. It is easy to show that the compiler described in §3.4 is still $\mathsf{CC}^{\sim}$. Assume that we want to satisfy a given notion of target noninterference after compilation, i.e., $\mathsf{W}{\downarrow} \models ANI^{\rho_{\mathbf{T}}}_{\phi_{\mathbf{T}}}$. Recall that the observational power of the target attacker, $\rho_{\mathbf{T}}$, is expressed as a property of sequences of values. To express the same property (or attacker) in the source, we have to abstract the way pairs of values are nested. For instance, the source attacker should not distinguish $\langle \mathsf{v}_1, \langle \mathsf{v}_2, \mathsf{v}_3 \rangle\rangle$ and $\langle\langle \mathsf{v}_1, \mathsf{v}_2 \rangle, \mathsf{v}_3\rangle$. In general (i.e., when $\overset{\circ}{\sim}$ is not the identity), this argument is valid only when $\phi_{\mathbf{T}}$ can be represented in the source. More precisely, $\phi_{\mathbf{T}}$ must consider as equivalent all target inputs that are related to the same source one, because in the source it is not possible to have a finer distinction of inputs. This intuitive correspondence can be formalized as follows:

**Theorem 4.3 (Target ANI by source ANI).** *Let* $\phi_{\mathbf{T}} \in uco(2^{\mathbf{Trace}^{\circ}_{\mathbf{T}}})$, $\rho_{\mathbf{T}} \in uco(2^{\mathbf{Trace}^{\bullet}_{\mathbf{T}}})$ *and* $\overset{\bullet}{\sim}$ *a total and surjective map from source outputs to target ones and assume that*

$$\forall \mathsf{s}\ \mathbf{t}.\ \mathsf{s}^{\circ} \overset{\circ}{\sim} \mathbf{t}^{\circ} \Rightarrow \phi_{\mathbf{T}}(\mathbf{t}^{\circ}) = \phi_{\mathbf{T}}(\tilde{\tau}^{\circ}(\mathsf{s}^{\circ})).$$

*If* $\cdot{\downarrow}$ *is a* $\mathsf{CC}^{\sim}$ *compiler and* $\mathsf{W}$ *satisfies* $ANI^{\rho^{\#}_{\mathsf{S}}}_{\phi^{\#}_{\mathsf{S}}}$, *then* $\mathsf{W}{\downarrow}$ *satisfies* $ANI^{\rho_{\mathbf{T}}}_{\phi_{\mathbf{T}}}$ *for*

$$\phi^{\#}_{\mathsf{S}} = \tilde{\sigma}^{\circ} \circ \phi_{\mathbf{T}} \circ \tilde{\tau}^{\circ}; \qquad\qquad \rho^{\#}_{\mathsf{S}} = \tilde{\sigma}^{\bullet} \circ \rho_{\mathbf{T}} \circ \tilde{\tau}^{\bullet}.$$

To wrap up the discussion about noninterference, the results presented in this section formalize and generalize some intuitive facts about compiler correctness and noninterference. Of course, they all place some restrictions on the shape of the noninterference instances that can be considered, because compiler correctness alone is in general not a strong enough criterion for dealing with many security properties [6, 17].

### 5 Trace-Relating Secure Compilation

So far we have studied compiler correctness criteria for whole, standalone programs. However, in practice, programs do not exist in isolation, but in a context where they interact with other programs, libraries, etc. In many cases, this context cannot be assumed to be benign and could instead behave maliciously to try to disrupt a compiled program.

Hence, in this section we consider the following *secure compilation* scenario: a source program is compiled and linked with an arbitrary target-level context, i.e., one that may not be expressible as the compilation of a source context. Compiler correctness does not address this case, as it does not consider arbitrary target contexts, looking instead at whole programs (empty context [33]) or well-behaved target contexts that behave like source ones (as in compositional compiler correctness [27, 30, 45, 57]).

To account for this scenario, Abate et al. [2] describe several secure compilation criteria based on the preservation of classes of (hyper)properties (e.g., trace properties, safety, hypersafety, hyperproperties, etc.) against arbitrary target contexts. For each of these criteria, they give an equivalent "property-free" criterion, analogous to the equivalence between TP and $\mathsf{CC}^{=}$. For instance, their *robust* trace property preservation criterion (RTP) states that, for any trace property $\pi$, if a source *partial* program $\mathsf{P}$ plugged into any context $\mathsf{C_S}$ satisfies $\pi$, then the compiled program $\mathsf{P}{\downarrow}$ plugged into any target context $\mathbf{C_T}$ satisfies $\pi$. Their equivalent criterion to RTP is RTC, which states that for any trace produced by the compiled program, when linked with any target context, there is a source context that produces the same trace. Formally (writing $C\,[P]$ to mean the whole program that results from linking partial program $P$ with context $C$) they define:

$$\mathsf{RTP} \equiv \forall \mathsf{P}.\ \forall \pi.\ (\forall \mathsf{C_S}.\ \forall t. \mathsf{C_S}\,[\mathsf{P}] \rightsquigarrow t \Rightarrow t \in \pi) \Rightarrow (\forall \mathbf{C_T}.\ \forall \mathbf{t}.\ \mathbf{C_T}\,[\mathsf{P}{\downarrow}] \rightsquigarrow t \Rightarrow t \in \pi);$$

$$\mathsf{RTC} \equiv \forall \mathsf{P}.\ \forall \mathbf{C_T}. \forall t. \mathbf{C_T}\,[\mathsf{P}{\downarrow}] \rightsquigarrow t \Rightarrow \exists \mathsf{C_S}.\ \mathsf{C_S}\,[\mathsf{P}] \rightsquigarrow t.$$

In the following we adopt the notation $P \models_R \pi$ to mean "$P$ *robustly* satisfies $\pi$," i.e., $P$ satisfies $\pi$ irrespective of the contexts it is linked with. Thus, we write more compactly:

$$\mathsf{RTP} \equiv \forall \pi.\ \forall \mathsf{P}.\ \mathsf{P} \models_{\mathsf{R}} \pi \Rightarrow \mathsf{P}{\downarrow} \models_{\mathbf{R}} \pi.$$

All the criteria of Abate et al. [2] share this flavor of stating the existence of some source context that simulates the behavior of any given target context, with some variations depending on the class of (hyper)properties under consideration. All these criteria are stated in a setting where source and target traces are the same. In this section, we extend their result to our trace-relating setting, obtaining trintarian views for secure compilation. Despite the similarities with §2, more challenges show up, in particular when considering the robust preservation of proper sub-classes of trace properties. For example, after application of $\tilde{\sigma}$ or $\tilde{\tau}$, a property may not be safety anymore, a crucial point for the equivalence with the property-free criterion for safety properties by Abate et al. [2]. We solve this by interpreting the class of safety properties as an *abstraction* of the class of all trace properties induced by a closure operator (§5.1). The remaining subsections provide example compilation chains satisfying our trace-relating secure compilation criteria for trace properties (§5.2) and for safety properties hypersafety (§5.3).

#### 5.1 Trace-Relating Secure Compilation: A Spectrum of Trinities

In this subsection we generalize many of the criteria of Abate et al. [2] using the ideas of §2. Before discussing how we solve the challenges for classes such as safety and hypersafety, we show the simple generalization of RTC to the trace-relating setting ($\mathsf{RTC}^{\sim}$) and its corresponding trinitarian view (Theorem 5.1):

**Theorem 5.1 (Trinity for Robust Trace Properties).** *For any trace relation $\sim$ and induced property mappings $\tilde{\tau}$ and $\tilde{\sigma}$, we have:* $\mathsf{RTP}^{\tilde{\tau}} \iff \mathsf{RTC}^{\sim} \iff \mathsf{RTP}^{\tilde{\sigma}}$, *where*

$$\mathsf{RTC}^{\sim} \equiv \forall \mathsf{P}\ \forall \mathbf{C_T}\ \forall \mathbf{t}.\ \mathbf{C_T}\,[\mathsf{P}{\downarrow}] \rightsquigarrow \mathbf{t} \Rightarrow \exists \mathsf{C_S}\ \exists \mathsf{s} \sim \mathbf{t}.\ \mathsf{C_S}\,[\mathsf{P}] \rightsquigarrow \mathsf{s};$$

$$\mathsf{RTP}^{\tilde{\tau}} \equiv \forall \mathsf{P}\ \forall \pi_{\mathsf{S}} \in 2^{\mathsf{Trace_S}}.\ \mathsf{P} \models_{\mathsf{R}} \pi_{\mathsf{S}} \Rightarrow \mathsf{P}{\downarrow} \models_{\mathbf{R}} \tilde{\tau}(\pi_{\mathsf{S}});$$

$$\mathsf{RTP}^{\tilde{\sigma}} \equiv \forall \mathsf{P}\ \forall \boldsymbol{\pi}_{\mathbf{T}} \in 2^{\mathbf{Trace}_{\mathbf{T}}}.\ \mathsf{P} \models_{\mathsf{R}} \tilde{\sigma}(\boldsymbol{\pi}_{\mathbf{T}}) \Rightarrow \mathsf{P}{\downarrow} \models_{\mathbf{R}} \boldsymbol{\pi}_{\mathbf{T}}.$$

Abate et al. [2] propose many more equivalent pairs of criteria, each preserving different classes of (hyper)properties, which we briefly recap now. For trace properties, they also have criteria that preserve safety properties plus their version of liveness properties. For hyperproperties, they have criteria that preserve hypersafety properties, subset-closed hyperproperties, and arbitrary hyperproperties. Finally, they define *relational* hyperproperties, which are relations between the behaviors of multiple programs for expressing, e.g., that a program always runs faster than another. For relational hyperproperties, they have criteria that preserve arbitrary relational properties, relational safety properties, relational hyperproperties and relational subset-closed hyperproperties. Roughly speaking, the security guarantees due to robust preservation of trace properties regard only protecting the integrity of the program from the context, the guarantees of hyperproperties also regard data confidentiality, and the guarantees of relational hyperproperties even regard code confidentiality. Naturally, these stronger guarantees are increasingly harder to enforce and prove.

While we have lifted the most significant criteria from Abate et al. [2] to our trinitarian view, due to space constraints we provide the formal definitions only for the two most interesting criteria. We summarize the generalizations of many other criteria in Figure 2, described at the end. Omitted definitions are available in the online appendix.

**Beyond Trace Properties: Robust Safety and Hyperproperty Preservation.** We detail robust preservation of safety properties and of arbitrary hyperproperties since they are both relevant from a security point of view and their generalization is interesting.

**Theorem 5.2 (Trinity for Robust Safety Properties ).** *For any trace relation* $\sim$ *and for the induced property mappings* $\tilde{\tau}$ *and* $\tilde{\sigma}$*, we have:*

$$\mathsf{RTP}^{Safe\circ\tilde{\tau}} \iff \mathsf{RSC}^{\sim} \iff \mathsf{RSP}^{\tilde{\sigma}}, \qquad \textit{where}$$

$$\mathsf{RSC}^{\sim} \equiv \forall \mathsf{P}\ \forall \mathbf{C_T}\ \forall \mathbf{t}\ \forall \mathbf{m} \leq \mathbf{t}.\mathbf{C_T}\,[\mathsf{P}{\downarrow}] {\rightsquigarrow} \mathbf{t} \Rightarrow \exists \mathsf{C_S}\ \exists \mathbf{t}' \geq \mathbf{m}\ \exists \mathsf{s} \sim \mathbf{t}'.\ \mathsf{C_S}\,[\mathsf{P}] {\rightsquigarrow} \mathsf{s};$$

$$\mathsf{RTP}^{Safe\circ\tilde{\tau}} \equiv \forall \mathsf{P} \forall \pi_{\mathsf{S}} \in 2^{\mathsf{Trace_S}}.\mathsf{P} \models_{\mathsf{R}} \pi_{\mathsf{S}} \Rightarrow \mathsf{P}{\downarrow} \models_{\mathbf{R}} (Safe \circ \tilde{\tau})(\pi_{\mathsf{S}});$$

$$\mathsf{RSP}^{\tilde{\sigma}} \equiv \forall \mathsf{P} \forall \boldsymbol{\pi}_{\mathbf{T}} \in \mathbf{Safety_T}.\mathsf{P} \models_{\mathsf{R}} \tilde{\sigma}(\boldsymbol{\pi}_{\mathbf{T}}) \Rightarrow \mathsf{P}{\downarrow} \models_{\mathbf{R}} \boldsymbol{\pi}_{\mathbf{T}}.$$

There is an interesting asymmetry between the last two characterizations above, which we explain now in more detail. $\mathsf{RSP}^{\tilde{\sigma}}$ quantifies over target safety properties, while $\mathsf{RTP}^{Safe\circ\tilde{\tau}}$ quantifies over *arbitrary* source properties, but imposes the composition of $\tilde{\tau}$ with $Safe$, which maps an arbitrary target property $\boldsymbol{\pi}_{\mathbf{T}}$ to the target safety property that best over-approximates $\boldsymbol{\pi}_{\mathbf{T}}$[8] (an analogous *closure* was needed for subset-closed hyperproperties in Theorem 2.11). More precisely, $Safe$ is a closure operator on target properties, with $\mathbf{Safety_T} = \left\{ Safe(\boldsymbol{\pi}_{\mathbf{T}}) \;\middle|\; \boldsymbol{\pi}_{\mathbf{T}} \in 2^{\mathbf{Trace_T}} \right\}$. The mappings

$$Safe \circ \tilde{\tau} : 2^{\mathsf{Trace_S}} \leftrightarrows \mathbf{Safety_T} : \tilde{\sigma}$$

determine a Galois connection between source trace properties and target safety properties, and ensure the equivalence $\mathsf{RTP}^{Safe\circ\tilde{\tau}} \iff \mathsf{RSP}^{\tilde{\sigma}}$ (). This argument generalizes to arbitrary closure operators on target properties () and on hyperproperties, as long as the corresponding class is a sub-class of subset-closed hyperproperties, and

[8] $Safe(\boldsymbol{\pi}_{\mathbf{T}}) = \cap \{ \boldsymbol{S}_{\mathbf{T}} \mid \boldsymbol{\pi}_{\mathbf{T}} \subseteq \boldsymbol{S}_{\mathbf{T}} \wedge \boldsymbol{S}_{\mathbf{T}} \in \mathbf{Safety_T} \}$ is the topological closure in the topology of Clarkson and Schneider [14], where safety properties coincide with the closed sets.

explains all but one of the asymmetries in Figure 2, the one that concerns the robust preservation of arbitrary hyperproperties:

**Theorem 5.3 (Weak Trinity for Robust Hyperproperties).** *For a trace relation* $\sim \subseteq \mathsf{Trace_S} \times \mathbf{Trace_T}$ *and induced property mappings* $\tilde{\sigma}$ *and* $\tilde{\tau}$, $\mathsf{RHC}^{\sim}$ *is equivalent to* $\mathsf{RHP}^{\tilde{\tau}}$*; moreover, if* $\tilde{\tau} \leftrightarrows \tilde{\sigma}$ *is a Galois insertion (i.e.,* $\tilde{\tau} \circ \tilde{\sigma} = id$*),* $\mathsf{RHC}^{\sim}$ *implies* $\mathsf{RHP}^{\tilde{\sigma}}$*, while if* $\tilde{\sigma} \leftrightarrows \tilde{\tau}$ *is a Galois reflection (i.e.,* $\tilde{\sigma} \circ \tilde{\tau} = id$*),* $\mathsf{RHP}^{\tilde{\sigma}}$ *implies* $\mathsf{RHC}^{\sim}$*,*

$$\textit{where } \mathsf{RHC}^{\sim} \equiv \forall \mathsf{P}\, \forall \mathbf{C_T}\, \exists \mathsf{C_S}\, \forall \mathbf{t}.\ \mathbf{C_T}[\mathsf{P}{\downarrow}] \rightsquigarrow \mathbf{t} \iff (\exists \mathsf{s} \sim \mathbf{t}.\ \mathsf{C_S}[\mathsf{P}] \rightsquigarrow \mathsf{s});$$

$$\mathsf{RHP}^{\tilde{\tau}} \equiv \forall \mathsf{P}\, \forall \mathsf{H_S}.\ \mathsf{P} \models_{\mathsf{R}} \mathsf{H_S} \Rightarrow \mathsf{P}{\downarrow} \models_{\mathbf{R}} \tilde{\tau}(\mathsf{H_S});$$

$$\mathsf{RHP}^{\tilde{\sigma}} \equiv \forall \mathsf{P}\, \forall \mathbf{H_T}.\ \mathsf{P} \models_{\mathsf{R}} \tilde{\sigma}(\mathbf{H_T}) \Rightarrow \mathsf{P}{\downarrow} \models_{\mathbf{R}} \mathbf{H_T}.$$

This trinity is *weak* since extra hypotheses are needed to prove some implications. While the equivalence $\mathsf{RHC}^{\sim} \iff \mathsf{RHP}^{\tilde{\tau}}$ holds unconditionally, the other two implications hold only under distinct, stronger assumptions. For $\mathsf{RHP}^{\tilde{\sigma}}$ it is still possible and correct to deduce a source obligation for a given target hyperproperty $\mathbf{H_T}$ when no information is lost in the the composition $\tilde{\tau} \circ \tilde{\sigma}$ (i.e., the two maps are a Galois *insertion*). On the other hand, $\mathsf{RHP}^{\tilde{\tau}}$ is a consequence of $\mathsf{RHP}^{\tilde{\sigma}}$ when no information is lost in composing in the other direction, $\tilde{\sigma} \circ \tilde{\tau}$ (i.e., the two maps are a Galois *reflection*).

**Navigating the Diagram.** For a given trace relation $\sim$, Figure 2 orders the generalized criteria according to their relative strength. If a trinity implies another (denoted by $\Rightarrow$), then the former provides stronger security for a compilation chain than the latter.

As mentioned, some property-full criteria regarding proper subclasses (i.e., subset-closed hyperproperties, safety, hypersafety, 2-relational safety and 2-relational hyperproperties) quantify over arbitrary (relational) (hyper)properties and compose $\tilde{\tau}$ with an additional operator. We have already presented the $Safe$ operator; other operators are $Cl_{\subseteq}$, $HSafe$, and $2rSafe$, which approximate the image of $\tilde{\tau}$ with a subset-closed hyperproperty, a hypersafety and 2-relational safety respectively.

As a reading aid, when quantifying over arbitrary trace properties we use the shaded blue as background color, we use the red when quantifying over arbitrary subset-closed hyperproperties and green for arbitrary 2-relational properties.

We now describe how to interpret the acronyms in Figure 2. All criteria start with R meaning they refer to robust preservation. Criteria for relational hyperproperties—here only arity 2 is shown—contain 2r. Next, criteria names spell the class of hyperproperties they preserve: H for hyperproperties, SCH for subset-closed hyperproperties, HS for hypersafety, T for trace properties, and S for safety properties. Finally, property-free criteria end with a C while property-full ones involving $\tilde{\sigma}$ and $\tilde{\tau}$ end with P. Thus, *robust (*R*) subset-closed hyperproperty-preserving (*SCH*) compilation (*C*)* is $\mathsf{RSCHC}^{\sim}$, *robust (*R*) two-relational (*2r*) safety-preserving (*S*) compilation (*C*)* is $\mathsf{R2rSC}^{\sim}$, etc.

### 5.2 Instance of Trace-Relating Robust Preservation of Trace Properties

This subsection illustrates trace-relating secure compilation when the target language has strictly more events than the source that target contexts can exploit to break security.

**Source and Target Languages.** The source and target languages used here are nearly identical expression languages, borrowing from the syntax of the source language of §3.3. Both languages add *sequencing* of expressions, two kinds of *output events*, and

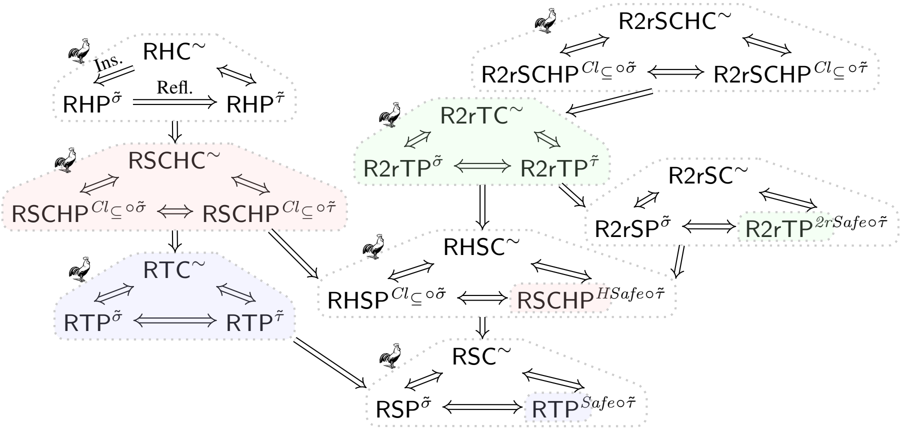

| | | |
|---|---|---|
| R robust | 2r 2-relational | |
| H hyperproperties | SCH subset-closed hyperproperties | HS hypersafety |
| T trace properties | S safety properties | |
| P property-full criterion | C property-free criterion based on $\sigma$ and $\tau$ | |

Fig. 2: Hierarchy of trinitarian views of secure compilation criteria preserving classes of hyperproperties and the key to read each acronym. Shorthands 'Ins.' and 'Refl.' stand for Galois Insertion and Reflection. The 🐓 symbol denotes trinities proven in Coq.

the expressions that generate them: $\mathsf{out_S}\ \mathsf{n}$ and $\mathbf{out_S}\ \mathbf{n}$ usable in source and target, respectively, and $\mathbf{out_T}\ \mathbf{n}$ usable only in the target, which is the only difference between source and target. The extra events in the target model the fact that the target language has an increased ability to perform certain operations, some of them potentially dangerous (such as writing to the hard drive), which cannot be performed by the source language, and against which source-level reasoning can therefore offer no protection.

Both languages and compilation chains now deal with partial programs, contexts and linking of those two to produce whole programs. In this setting, a whole program is the combination of a *main expression* to be evaluated and a set of *function definitions* (with distinct names) that can refer to their argument symbolically and can be called by the main expression and by other functions. The set of functions of a whole program is the union of the functions of a partial program and a context; the latter also contains the main expression. The extensions of the typing rules and the operational semantics for whole programs are unsurprising and therefore elided. The trace model also follows closely that of §3.3: it consists of a list of *regular events* (including the new outputs) terminated by a *result event*. Finally, a partial program and a context can be linked into a whole program when their functions satisfy the requirements mentioned above.

**Relating Traces.** In the present model, source and target traces differ only in the fact that the target draws (regular) events from a strictly larger set than the source, i.e., $\mathbf{\Sigma_T} \supset \Sigma_\mathsf{S}$. A natural relation between source and target traces essentially maps to a given target trace $\mathbf{t}$ the source trace that erases from $\mathbf{t}$ those events that exist only at the target level. Let $\mathbf{t}|_{\Sigma_\mathsf{S}}$ indicate trace $\mathbf{t}$ filtered to retain only those elements included in alphabet $\Sigma_S$. We define the trace relation as:

$$s \sim t \quad \equiv \quad s = t|_{\Sigma_S}.$$

In the opposite direction, a source trace $s$ is related to many target ones, as any target-only events can be inserted at any point in $s$. The induced mappings for $\sim$ are:

$$\tilde{\tau}(\pi_S) = \{t \mid \exists s.\, s = t|_{\Sigma_S} \wedge s \in \pi_S\}\,; \quad \tilde{\sigma}(\pi_T) = \{s \mid \forall t.\, s = t|_{\Sigma_S} \Rightarrow t \in \pi_T\}\,.$$

That is, the target guarantee of a source property is that the target has the same source-level behavior, sprinkled with arbitrary target-level behavior. Conversely, the source-level obligation of a target property is the aggregate of those source traces all of whose target-level enrichments are in the target property.

Since $R^S$ and $\mathbf{R^T}$ are very similar, it is simple to prove that the identity compiler $(\cdot\!\downarrow)$ from $R^S$ to $\mathbf{R^T}$ is secure according to the trace relation $\sim$ defined above.

**Theorem 5.4** **($\cdot\!\downarrow$ is Secure 🐓).** $\cdot\!\downarrow$ *is* $\mathsf{RTC}^{\sim}$.

#### 5.3 Instances of Trace-Relating Robust Preservation of Safety and Hypersafety

To provide examples of cross-language trace-relations that preserve safety and hypersafety properties, we show how existing secure compilation results can be interpreted in our framework. This indicates how the more general theory developed here can already be instantiated to encompass existing results, and that existing proof techniques can be used in order to achieve the secure compilation criteria we define.

For the preservation of safety, Patrignani and Garg [50] study a compiler from a typed, concurrent WHILE language to an untyped, concurrent WHILE language with support for memory capabilities. As in §3.3, their source has bools and nats while their target only has **nat**s. Additionally, their source has an ML-like memory (where the domain is locations $\ell$) while their target has an assembly-like memory (where the domain is natural numbers $\mathbf{n}$). Their traces consider context-program interactions and as such they are concatenations of call and return actions with parameters, which can include booleans as well as locations. Because of the aforementioned differences, they need a cross-language relation to relate source and target actions.

Besides defining a relation on traces (i.e., an instance of $\sim$), they also define a relation between source and target safety properties. They provide an instantiation of $\tau$ that maps all safe source traces to the related target ones. This ensures that no additional target trace is introduced in the target property, and source safety properties are mapped to target safety ones by $\tau$. Their compiler is then proven to generate code that respects $\tau$, so they achieve a variation of $\mathsf{RTP}^{Safe \circ \tilde{\tau}}$.

Concerning the preservation of hypersafety, Patrignani and Garg [49] consider compilers in a reactive setting where traces are sequences of input ($\alpha?$) and output ($\alpha!$) actions. In their setting, traces are different between source and target, so they define a cross-language relation on actions that is total on the source actions and injective. Additionally, their set of target output actions is strictly larger than the source one, as it includes a special action $\surd$, which is how compiled code must respond to invalid target inputs (i.e., receiving a **bool** when a **nat** was expected). Starting from the relation on actions, they define **TPC**, which is an instance of what we call $\tau$. Informally, given a set of source traces, **TPC** generates all target traces that are related (pointwise) to a source trace. Additionally, it generates all traces with interleavings of undesired inputs $\boldsymbol{\alpha?}$ followed by $\surd$ as long as removing $\boldsymbol{\alpha?}\surd$ leaves a trace that relates to the source trace.

TPC preserves hypersafety across languages, i.e., it is an instance of $\mathsf{RSCHP}^{HSafe \circ \tilde{\tau}}$ mapping source hypersafety to target hypersafety (and safety to safety).

## 6 Related Work

We already discussed how our results relate to some existing work in correct compilation [33, 58] and secure compilation [2, 49, 50]. We also already mentioned that most of our definitions and results make no assumptions about the structure of traces. One result that relies on the structure of traces is Theorem 5.2, which involves some *finite prefix* $m$, suggesting traces should be some sort of sequences of events (or states), as customary when one wants to refer to safety properties [14]. It is however sufficient to fix a topology on properties where safety properties coincide with closed sets [46]. Even for reasoning about safety, hypersafety, or arbitrary hyperproperties, traces can therefore be values, sequences of program states, or of input output events, or even the recently proposed *interaction trees* [62]. In the latter case we believe that the compilation from IMP to ASM proposed by Xia et al. [62] can be seen as an instance of $\mathsf{HC}^{\sim}$, for the relation they call "trace equivalence."

**Compilers Where Our Work Could Be Useful.** Our work should be broadly applicable to understanding the guarantees provided by many verified compilers. For instance, Wang et al. [61] recently proposed a CompCert variant that compiles all the way down to machine code, and it would be interesting to see if the model at the end of §3.1 applies there too. This and many other verified compilers [12, 29, 42, 56] beyond CakeML [58] deal with resource exhaustion and it would be interesting to also apply the ideas of §3.2 to them. Hur and Dreyer [27] devised a correct compiler from an ML language to assembly using a cross-language logical relation to state their CC theorem. They do not have traces, though were one to add them, the logical relation on values would serve as the basis for the trace relation and therefore their result would attain $\mathsf{CC}^{\sim}$.

Switching to more informative traces capturing the interaction between the program and the context is often used as a proof technique for secure compilation [2, 28, 48]. Most of these results consider a cross-language relation, so they probably could be proved to attain one of the criteria from Figure 2.

**Generalizations of Compiler Correctness.** The compiler correctness definition of Morris [41] was already general enough to account for trace relations, since it considered a translation between the semantics of the source program and that of the compiled program, which he called "decode" in his diagram, reproduced in Figure 3 (left). And even some of the more recent compiler correctness definitions preserve this kind of flexibility [51]. While $\mathsf{CC}^{\sim}$ can be seen as an instance of a definition by Morris [41], we are not aware of any prior work that investigated the preservation of properties when the "decode translation" is neither the identity nor a bijection, and source properties need to be re-interpreted as target ones and vice versa.

**Correct Compilation and Galois Connections.** Melton et al. [38] and Sabry and Wadler [55] expressed a strong variant of compiler correctness using the diagram of Figure 3 (right) [38, 55]. They require that compiled programs *parallel* the computation steps of the original source programs, which can be proven showing the existence of a *decompilation* map $\#$ that makes the diagram commute, or equivalently, the existence of an adjoint for $\downarrow$ ($W \leq W' \iff W \twoheadrightarrow W'$ for both source and target). The

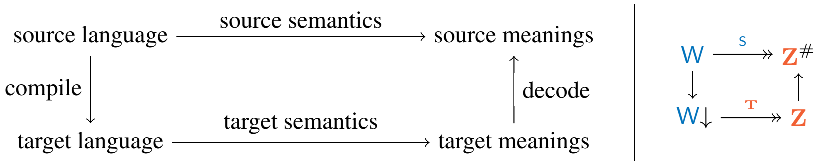

Fig. 3: Morris's [41] (left) and Melton et al.'s [38] and Sabry and Wadler's [55] (right)

"parallel" intuition can be formalized as an instance of $\mathsf{CC}^{\sim}$. Take source and target traces to be finite or infinite sequences of program states (maximal trace semantics [15]), and relate them exactly like Melton et al. [38] and Sabry and Wadler [55].

**Translation Validation.** Translation validation is an important alternative to proving that all runs of a compiler are correct. A variant of $\mathsf{CC}^{\sim}$ for translation validation can simply be obtained by specializing the definition to a particular $\mathsf{W}$, and one can obtain again the same trinitarian view. Similarly for our other criteria, including our extensions of the secure compilation criteria of Abate et al. [2], which Busi et al. [10] seem to already be considering in the context of translation validation.

### 7 Conclusion and Future Work

We have extended the property preservation view on compiler correctness to arbitrary trace relations, and believe that this will be useful for understanding the guarantees various compilers provide. An open question is whether, given a compiler, there exists a most precise $\sim$ relation for which this compiler is correct. As mentioned in §1, every compiler is $\mathsf{CC}^{\sim}$ for some $\sim$, but under which conditions is there a most precise relation? In practice, more precision may not always be better though, as it may be at odds with compiler efficiency and may not align with more subjective notions of usefulness, leading to tradeoffs in the selection of suitable relations. Finally, another interesting direction for future work is studying whether using the relation to Galois connections allows to more easily compose trace relations for different purposes, say, for a compiler whose target language has undefined behavior, resource exhaustion, and side-channels. In particular, are there ways to obtain complex relations by combining simpler ones in a way that eases the compiler verification burden?

**Acknowledgements.** We thank Akram El-Korashy and Amin Timany for participating in an early discussion about this work and the anonymous reviewers for their valuable feedback. This work was in part supported by the European Research Council under ERC Starting Grant SECOMP (715753), by the German Federal Ministry of Education and Research (BMBF) through funding for the CISPA-Stanford Center for Cybersecurity (FKZ: 13N1S0762), by DARPA grant SSITH/HOPE (FA8650-15-C-7558) and by UAIC internal grant 07/2018.
